# The Potential Application of Chinese Medicine in Liver Diseases: A New Opportunity

**DOI:** 10.3389/fphar.2021.771459

**Published:** 2021-11-04

**Authors:** Ke Fu, Cheng Wang, Cheng Ma, Honglin Zhou, Yunxia Li

**Affiliations:** State Key Laboratory of Southwestern Chinese Medicine Resources, Key Laboratory of Standardization for Chinese Herbal Medicine, Ministry of Education, School of Pharmacy, Chengdu University of Traditional Chinese Medicine, Chengdu, China

**Keywords:** liver diseases, natural agents, toxicity, clinical trials, potential application, Chinese medicine

## Abstract

Liver diseases have been a common challenge for people all over the world, which threatens the quality of life and safety of hundreds of millions of patients. China is a major country with liver diseases. Metabolic associated fatty liver disease, hepatitis B virus and alcoholic liver disease are the three most common liver diseases in our country, and the number of patients with liver cancer is increasing. Therefore, finding effective drugs to treat liver disease has become an urgent task. Chinese medicine (CM) has the advantages of low cost, high safety, and various biological activities, which is an important factor for the prevention and treatment of liver diseases. This review systematically summarizes the potential of CM in the treatment of liver diseases, showing that CM can alleviate liver diseases by regulating lipid metabolism, bile acid metabolism, immune function, and gut microbiota, as well as exerting anti-liver injury, anti-oxidation, and anti-hepatitis virus effects. Among them, Keap1/Nrf2, TGF-β/SMADS, p38 MAPK, NF-κB/IκBα, NF-κB-NLRP3, PI3K/Akt, TLR4-MyD88-NF-κB and IL-6/STAT3 signaling pathways are mainly involved. In conclusion, CM is very likely to be a potential candidate for liver disease treatment based on modern phytochemistry, pharmacology, and genomeproteomics, which needs more clinical trials to further clarify its importance in the treatment of liver diseases.

## Introduction

Chinese medicine (CM) is an effective drug treatment system with a history of thousands of years. It is used for disease prevention, treatment and diagnosis. CM is characterized by individualized adjustment of multiple components and multiple targets, which makes the body change from an abnormal state to a normal state (Wang et al., 2018). It has made an indelible contribution to human health and is considered a potential natural source of therapeutic drugs (Hesketh and Zhu, 1997; Chan and Ng, 2020). For example, Tu won the 2015 Nobel Prize for discovering and developing artemisinin in *Artemisia annua* Linn. It is a clear example to prove the therapeutic potential of CM and is of great significance to the continued development of the field (Tu, 2016). Besides, this field has huge and undeveloped resources. Screening and providing effective monomer chemicals are important means of CM to promote the development of medicine in the world (Wang et al., 2018).

Liver diseases are serious diseases threatening the whole human health, mainly including metabolic associated fatty liver disease (MAFLD), alcoholic liver disease (ALD), chronic viral hepatitis (e.g., hepatitis B virus (HBV) and hepatitis C virus (HCV) infections), autoimmune hepatitis, hepatic schistosomiasis, drug-induced liver injury, liver cirrhosis (LC), hepatocellular carcinoma (HCC), and so on (Li, Q. et al., 2018; Wang et al., 2014). China has the highest incidence of liver diseases in the world, and about 300,000–400,000 people die from various liver diseases each year. According to the data, MAFLD, HBV and ALD are the three most common liver diseases in China, with the incidence of 49.3, 22.9 and 14.8% respectively (Wang et al., 2014).

At present, CM has shown significant efficacy in the treatment of liver diseases, such as *Rheum palmatum* L. (Jin et al., 2005; Yang et al., 2012; Neyrinck et al., 2017), *Silybum marianum* (L.) Gaertn. (Alaca et al., 2017; Jindal et al., 2019), and *Sophora flavescens* Ait. (Yang et al., 2018; Yim et al., 2019). Furthermore, liver diseases are various, and the course of each disease is also different. Fortunately, CM can effectively treat a variety of liver diseases, and it has played an important role in the prevention and treatment of liver diseases. For example, *Zingiber officinale* and *Glycyrrhiza uralensis* Fischer can effectively treat ALD and MAFLD (Jung et al., 2016; Kandeil et al., 2019), and *Rhizoma Coptidis* can be used in the treatment of hepatitis virus (Hung et al., 2018). For more serious liver diseases, such as liver cirrhosis and liver cancer, *Salvia offificinalis* L. and *Portulaca oleracea* L. have shown good effects (Guoyin et al., 2017; Jiang, Y. et al., 2017). Besides, according to relevant records, the variety of CM commonly used in the treatment of liver diseases is up to 90 kinds (Wu, 2001). It can be seen that the resources of CM for the treatment of liver diseases are rich and valuable, which is worthy of further research and development.

In this review, we collected relevant literature in recent 6 years (2015–2020) through CNKI, PubMed, ScienceDirect and Google academic, and analyzed the application, toxicology and clinical data of CM and their related compounds, aiming to dig out more CM with potential biological activities for liver diseases, and promote their application value in the treatment of liver diseases, further providing relevant reference for the clinical application CM.

## Characteristics of Several Important Liver Diseases

### The Three Most Common Liver Diseases in China

#### MAFLD

MAFLD is a clinical syndrome characterized by hepatocyte steatosis and increased lipid deposition with the exception of alcohol and other clear liver-damaging factors (Mantovani et al., 2019). It is associated with obesity, insulin resistance, type 2 diabetes mellitus, hypertension, hyperlipidemia, and metabolic syndrome (Younossi, 2019). MAFLD is a broad umbrella term for a range of liver disorders, from non-alcoholic fatty liver (NAFL) to non-alcoholic steatohepatitis (NASH). It is called NAFL if it is only steatosis (fatty liver) and NASH if there is severe inflammation and liver cell damage (steatohepatitis). The course of MAFLD is complex and variable, which can lead to cirrhosis and liver cancer in severe cases (Friedman et al., 2018).

The pathogenesis of MAFLD mainly includes abnormal lipid metabolism, oxidative stress, inflammasome activation, insulin resistance, mitochondrial dysfunction, and genetic determinants (Buzzetti et al., 2016). Abnormal lipid metabolism in hepatocytes is the initial factor for MAFLD. When the number of fatty acids entering the liver is greater than their oxidation and secretion, the lipid accumulates in the liver, resulting in hepatic lipid deposition (Onyekwere et al., 2015), which leads directly to MAFLD. Furthermore, excessive lipid deposition further aggravates tissue damage by promoting the production of reactive oxygen species (ROS) and a series of pathological changes, such as the peroxidation of cells themselves, the release of pro-inflammatory factors and the infiltration of inflammatory cells, damaged hepatocytes activate the nuclear factor kappa-B (NF-κB) pathway, thus inducing the production of proinflammatory cytokine tumor necrosis factor-α (TNF-α) and interleukin-1β/-6 (IL-1β, IL-6) (Buzzetti et al., 2016; Xiao et al., 2020). These inflammatory factors can not only induce the activation of astrocytes and the remodeling of cell matrix, but also accelerate the progression of the disease by promoting insulin resistance. In addition, MAFLD is strongly associated with gut microbes, some of which carry genes that ferment dietary sugars into ethanol. When released into the bloodstream, they will increase oxidative stress and inflammation in the liver. In the liver, alcohol dehydrogenase metabolizes ethanol into toxic acetaldehyde, which forms adducts with proteins and other molecules in the cell because of its electrophilic properties, resulting in the loss of hepatocyte structure and function (Kolodziejczyk et al., 2019).

#### HBV Infection

HBV, a part of the *Hepadnaviridae* family, consists of nucleocapsid, envelope, and three complete membrane proteins (Seitz et al., 2007), which is a partially double-stranded and non-cytopathic DNA virus. The virus replicates the DNA by reverse transcription of the pre-RNA genome and has many serological markers such as HBsAg and anti-HBs, HBeAg and anti-HBe, and anti-HBc IgM and IgG (Trépo et al., 2014; Hu and Liu, 2017). HBV is the most common chronic virus in the world. Infected cells produce covalently closed circular DNA intermediates and integrated sequences that act as transcription templates for viral proteins (Fanning et al., 2019). HBV is transmitted through a number of routes, but mainly in the form of blood and body fluids, including perinatal and mother-to-child transmission, as well as sexual and extraintestinal patterns (Yuen et al., 2018).

At present, vaccination is still the most effective tool to prevent HBV infection, but there are also other therapeutic approaches, such as antiviral drugs that directly act on virus replication (interferon) and immune modulators (including reverse-transcriptase inhibitors, primarily a nucleoside or nucleotide analogue) (Yuen et al., 2018). These treatments can effectively inhibit HBV replication, but the disadvantages are the long-term medication and side effects. In addition, HBV infection can lead to chronic hepatitis and a series of complications, and studies have shown that HBV may persist in the body even after the infected person has fully recovered (Rehermann et al., 1996; Shi and Zheng, 2020). If immunosuppression-mediated host immune control is weakened, or several therapies and drugs have a direct effect on HBV replication, HBV may be reactivated (Shi and Zheng, 2020). Therefore, it is urgent to find a more effective HBV therapy to ensure the health of all human beings.

#### ALD

ALD refers to hepatocyte necrosis and destruction of normal liver function under the action of ethanol for a long time, which is a series of liver diseases including fatty liver, alcoholic hepatitis, cirrhosis, and its complications (such as ascites, portal hypertension-related bleeding, hepatic encephalopathy, and HCC) (Singal et al., 2018). The disease initially presents as alcoholic fatty liver disease, then gradually develops into alcoholic cirrhosis, even extensive hepatocyte necrosis, eventually inducing liver failure (Penny, 2013; Hu et al., 2019).

Sustained large quantity of alcohol stimulation is the primary factor of ALD. The pathogenesis is complicated and varied, mainly related to genetics, oxidative stress, hepatic steatosis, hepatic inflammation, and so on (2018). There is some evidence that aldehyde dehydrogenase2*2 and alcohol dehydrogenase 1B*3 alleles are closely related to alcoholic liver disease, and they can have some kind of chemical reaction with alcohol to achieve rapid metabolism (Agrawal and Bierut, 2012; Dodge et al., 2014); transmembrane 6 superfamily member 2 gene mutation can lead to the accumulation of liver fat, so that the disease will develop into a bad situation (2018); patatinlike phospholipase domain-containing protein 3, which mediates triglyceride hydrolysis in adipocytes, is closely related to lipid metabolism in the liver, but the mechanism of how it affects ALD is unclear (Salameh et al., 2015; BasuRay et al., 2017). At the same time, membrane-bound O-acetyltransferase domain-containing protein 7 is also an important genetic material related to ALD, but its mechanism is not clear (2018).

Oxidative stress plays a crucial role in the pathogenesis of ALD. In biological systems, free radicals include oxygen free radicals and nitrogen free radicals, among which oxygen free radicals and non-free radicals such as hypochlorite and ozone are called ROS. Under normal circumstances, the body contains antioxidants (such as superoxide dismutase (SOD), catalase, glutathione (GSH), glutathione peroxidase, glutathione transferase, heme oxygenase bilirubin etc.) and ROS in a state of balance, which are not harmful to the human body (Li et al., 2015). But in the case of long-term alcohol abuse, the reduction in the level or activity of antioxidants in the body causes oxidative stress. Alcohol may also increase the level of ROS. For example, ROS and nicotinamide adenine dinucleotide (NADH) are produced when ethanol is oxidized to acetaldehyde by alcohol dehydrogenase in the liver. Acetaldehyde is oxidized to acetic acid in mitochondria, which stimulates the body to produce large amounts of ROS (Li et al., 2014). NADH also interferes with the mitochondrial electron transport system and promotes ROS production (Ceni et al., 2014). Alcohol can also activate the NAD (P) H oxidase in hepatocytes, leading to an increase in the production of superoxide (Kalyanaraman, 2013). There is also evidence that another important pathophysiological mechanism of ALD is the interaction between endotoxin and Kupffer cells (KCs). Long-term high alcohol intake can induce low levels of intestinal endotoxemia, and increase intestinal permeability, causing Gram-negative bacteria to enter the hepatic portal circulation to suppress immune function (Mello et al., 2008; Gao and Liu, 2016). KCs recognize and clear gut-derived endotoxins, and promote oxidative stress and inflammatory response through their interaction (Yang and Wei, 2017).

### Other Liver Diseases

#### HCV Infection

Hepatitis C is an infectious disease caused by HCV. HCV is an RNA virus, 45–65 nm in diameter, encapsulated in a lipid bilayer, belonging to the *Flaviviridae* family (Manns et al., 2017). HCV enters its target cells by a variety of host factors, including CD81, low-density lipoprotein receptor, dendritic cell-specific ICAM-grabbing non-integrin, claudin-1, and occludin. Among the different types of liver diseases, HCV is unique in requiring liver specific microRNA-122 replication (Luna et al., 2015). In addition, the genotypes of HCV are very rich. By the culture, analysis and identification of HCV strains isolated from all parts of the world, seven major HCV genotypes were found, namely 1–7 (Manns et al., 2017). Genotype 1 is the most prevalent in the world, including 83.4 million cases (46.2% of all HCV cases), about a third of which are in East Asia. Genotype 3 ranks second in the world (54.3 million, 30.1%), genotype 2, 4 and 6 account for 22.8% of all cases, and genotype 5 accounts for less than 1% of the remaining cases (Messina et al., 2015).

HCV transmission is most commonly associated with direct percutaneous exposure to blood *via* blood transfusions, health-care-related injections, and injecting drug use (Spearman et al., 2019). Alcohol is also a common cofactor for HCV infection, and alcohol use is more strongly associated with the progression of liver fibrosis (Poynard et al., 1997). Secondly, HCV infection can induce the abnormal expression of two host microRNAs (miR-208b and miR-499a-5p) encoded by myosin genes in hepatocytes. MiR-208b and miR-499a-5p inhibit type I IFN signal transduction in infected hepatocytes by directly down-regulating type I IFN receptor expression (Jarret et al., 2016). In addition, chronic HCV infection can also lead to liver fibrosis, cirrhosis, hepatocellular carcinoma and other serious complications.

#### LC

LC is a pathological stage characterized by diffuse fibrosis, pseudolobules formation, and intrahepatic and extrahepatic vascular proliferation (He and Liu, 2021). It is one of the main causes of death in patients with liver diseases all over the world, and also the final result of the development of a variety of acute and chronic liver diseases. LC shows symptoms such as portal hypertension and liver dysfunction. At present, the diagnosis of LC mainly depends on the imaging of irregular nodular liver by ultrasound, CT or MRI and the evaluation of liver synthesis function. In clinical practices, LC is considered as an end-stage manifestation of liver pathology with a high mortality without liver transplantation treatment (Tsochatzis et al., 2014; Zhou et al., 2014). But liver transplantation requires a lot of ligands and money, which is not an easy thing to solve, so CM has become a more effective approach.

The pathological pathway of LC is very complicated, but the research has shown that it is closely related to the expression of some cells on the wall of hepatic sinus. Hepatic sinus walls are composed of three kinds of non-parenchymal cells (liver sinusoidal endothelial cells (LSECs), KCs and hepatic stellate cells (HSCs)), which are involved in the development of LC (Zhou et al., 2014). In non-diseased liver, HSCs are located in the subendothelial space of Disse and are primarily involved in the storage of retinoic acid, but HSC is activated in the area of liver injury (Friedman, 1993; Hernandez-Gea and Friedman, 2011). In this activated phenotype, HSC is the main source of collagen and non-collagen matrix proteins in fibrosis. Related studies have shown that LSECs can secrete the cytokine IL-33 to activate HSCs and promote fibrosis (Marvie et al., 2010). Secondly, the exfoliation and capillarization of LSECs were proved to be the main contributing factors of liver dysfunction in cirrhosis (Yokomori et al., 2012). Finally, KCs can mediate liver inflammation to aggravate liver damage and fibrosis (López-Navarrete et al., 2011). Cytokines such as platelet-derived growth factor, transforming growth factor-β (TGF-β), TNF-α, and Interferon also play a crucial role in the pathogenesis of liver fibrosis and cirrhosis (Zhou et al., 2014). It is worth mentioning that if a patient has been diagnosed with ALD, concomitant chronic hepatitis B or C infection will directly aggravate the liver injury, leading to more frequent and rapid occurrence of cirrhosis (Poynard et al., 1997).

#### HCC

HCC is the most common form of liver cancer, accounting for 90% of the total cases of liver cancer. Among the various chronic liver diseases, HCC is the final stage of the disease in some patients with LC. About 80% of HCC patients have the pathological basis of LC, and the rate of HCC in patients with cirrhosis disease base in the short-term can be 5–30% (El-Serag, 2012). HBV and HCV are major risk factors for the development of HCC (Llovet et al., 2021). Others include exposure to aflatoxin, excessive drinking, smoking, diabetes, and knowledge of other risk factors such as MAFLD has been gradually recognized (Forner et al., 2012; Forner et al., 2018). The high incidence of HCC is concentrated in developing countries such as China, mainly due to chronic HBV infection (Jemal et al., 2011). Until now, there has been no nationwide cancer screening in China. Once a patient develops HCC, not only does the patient face tremendous pain from radiation therapy, but the improvement in survival rates is very limited, if more potential anti-cancer drugs can be tapped from the CM system, it will be beneficial to HCC patients.

**FIGURE 1:**
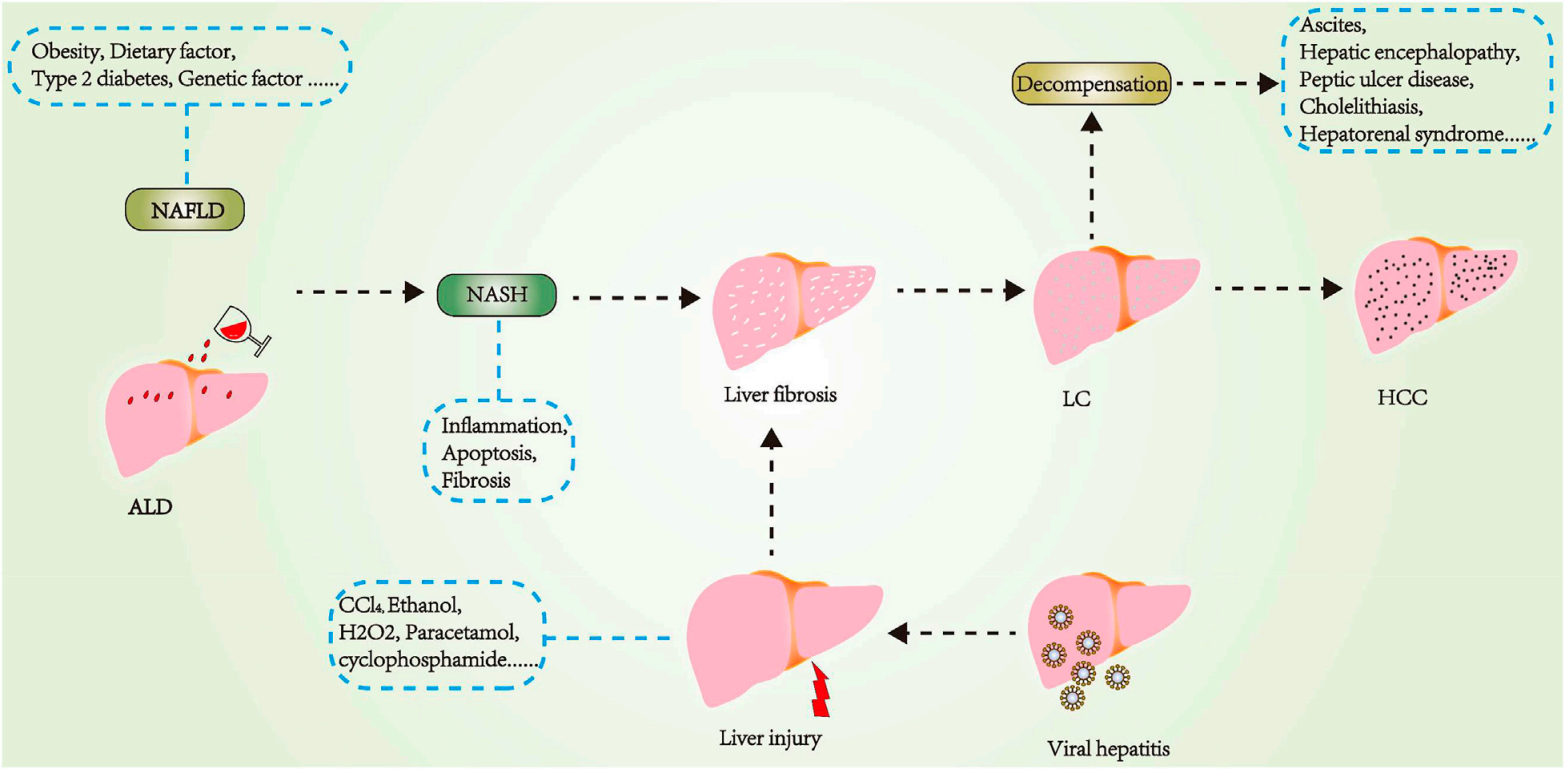
Main pathogenesis of important liver diseases.

Figure 1 is a map of the major pathogenesis of some important liver diseases.

### Pharmacological Effects of CM for Management of Liver Disease

**FIGURE 2:**
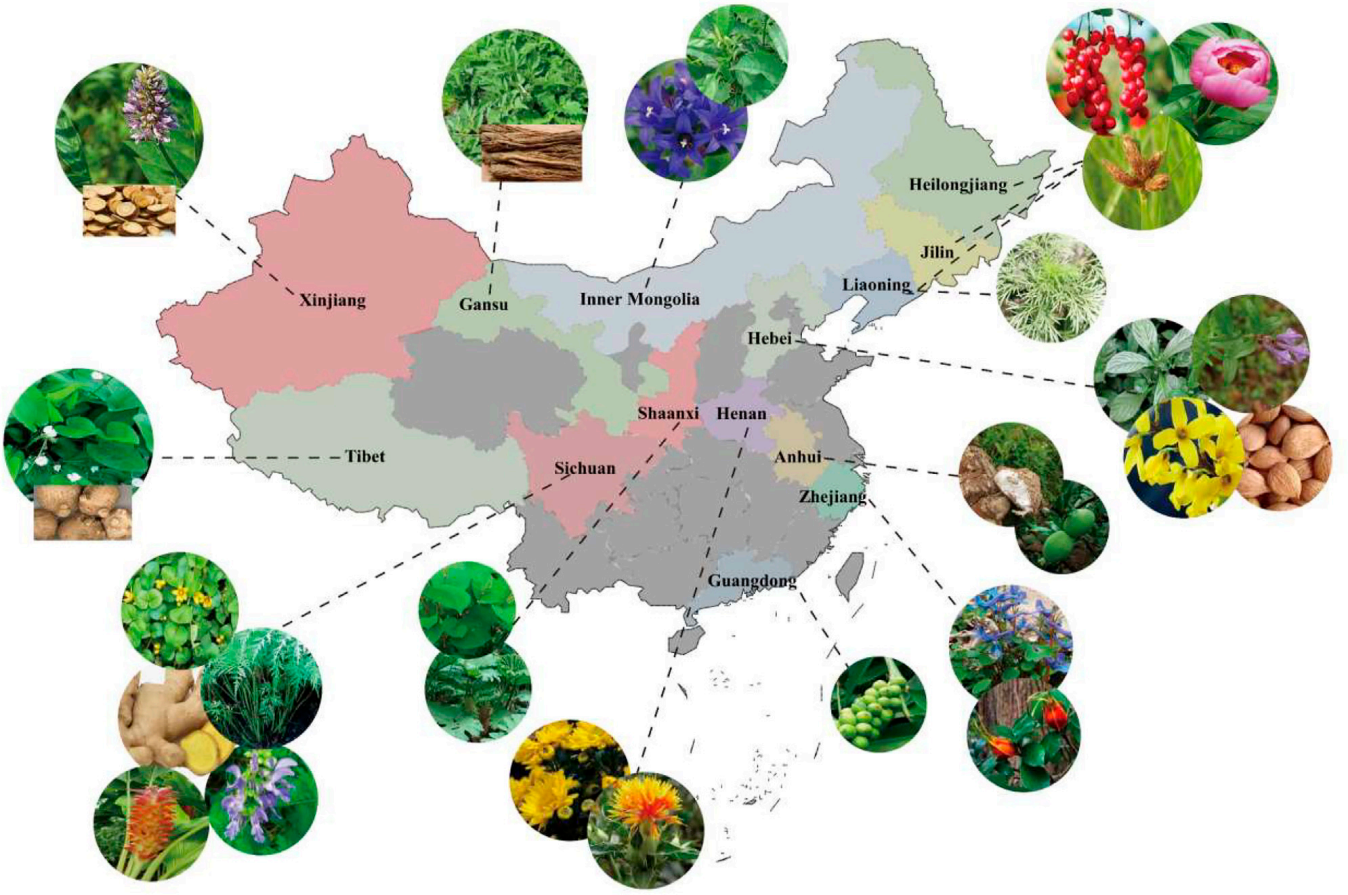
Distribution of some Chinese medicine for liver diseases in main producing areas (also named “Daodi” producing area). Shaanxi: *Rheum palmatum* L., *Polygonum cuspidatum* Sieb.et Zucc.; Sichuan: *Salvia miltiorrhiza* Bunge., *Zingiber officinale* Rosc., *Ligusticum chuanxiong* Hort., *Curcuma wenyujin* Y. H. Chen et C. Ling, *Lysimachia christinae* Hance; Gansu: *Angelica sinensis* (Oliv.) Diels; Tibet: *Alisma orientalis* (Sam.) Juzep.; Hebei: *Prunus persica* (L.) Batsch, *Forsythia suspensa* (Thunb.) Vahl, *Isatis indigotica* Fort., *Scutellaria baicalensis* Georgi; Henan: *Carthamus tinctorius* L., *Chrysanthemum morifolium* Ramat.; Zhejiang: *Gardenia jasminoides* Ellis, *Corydalis yanhusuo* W. T. Wang; Liaoning: *Artemisia scoparia* Waldst. et Kit.; Guangdong: *Alpinia oxyphylla* Miq.; Anhui: *Chaenomeles speciosa* (Sweet) Nakai, *Poria cocos* (Schw.) Wolf; Xinjiang: *Glycyrrhiza uralensis* Fisch.; Inner Mongolia: *Gentiana scabra* Bunge, *Isatis indigotica* Fort.; Dongbei (Heilongjiang, Jilin, Liaoning): *Schisandra chinensis* (Turcz.) Baill., *Paeonia lactiflora* Pall., *Sparganium stoloniferum* Buch-Ham.

**FIGURE 3:**
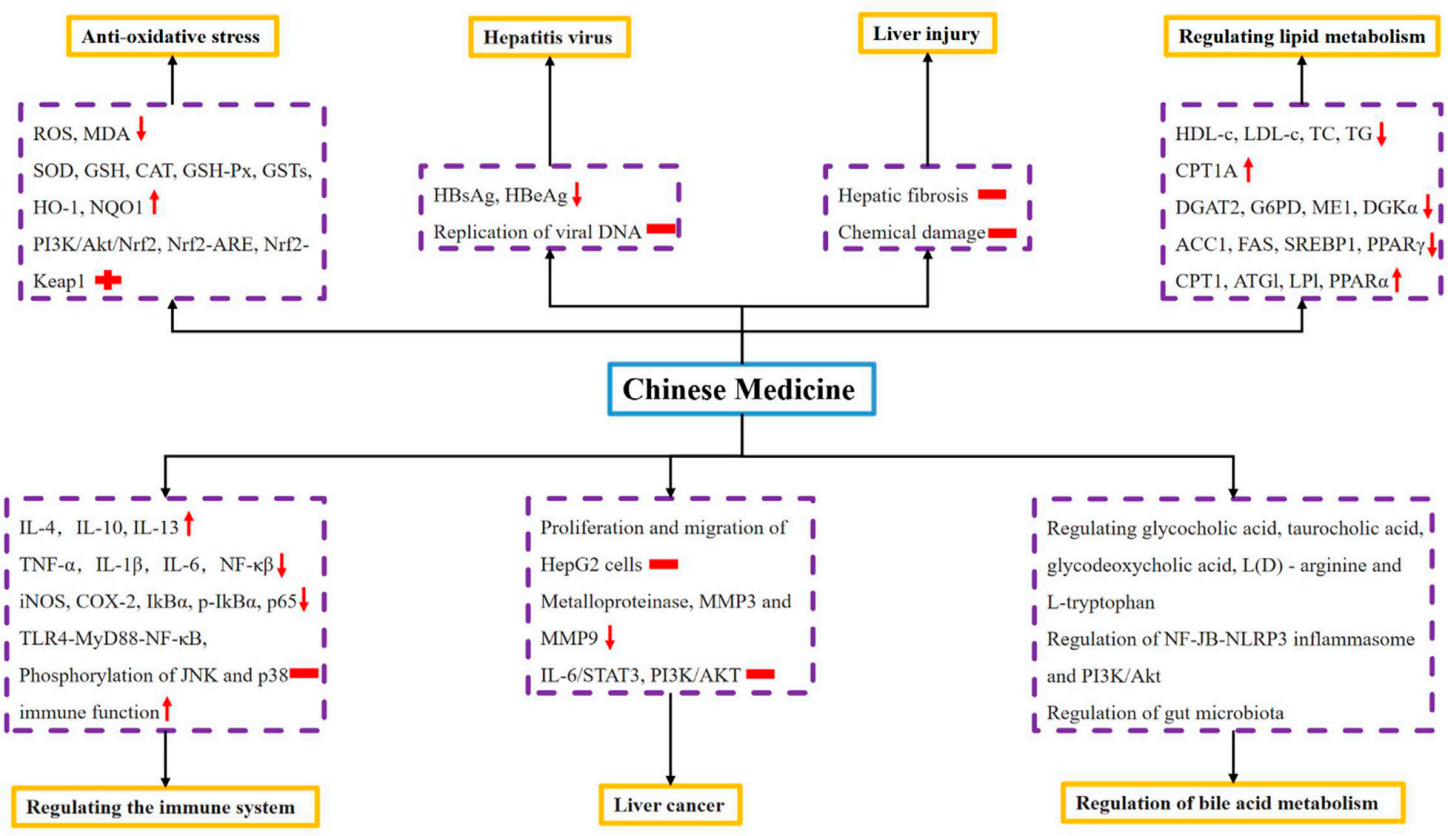
Pharmacological effects of Chinese medicine on liver diseases.

There are abundant varieties of natural CM resources in China, which is worthy of further development and utilization. For example, Figure 2 only shows the distribution of some CM for liver disease in the main producing area (also named “Daodi” producing area). Among them, many of the common CM have shown anti-liver disease activity, *see* Table 1. In addition, the pharmacological effects of CM on liver disease are summarized in Figure 3.

**TABLE 1:** Some of the Chinese medicine used for the treatment of liver diseases are described in the standard and their biological activities.

No	Latin name	English name	Family	Used part	Types of liver diseases that can be treated recorded in the standard	Reported biological activities associated with liver diseases
1	*Rheum palmatum *L	Rhei Radix et Rhizoma	Polygonaceae	Root and rhizome	Damp-heat jaundice^a^; Acute infectious hepatitis^b^	Regulating gut microbiota Neyrinck et al. (2017), protective effect on high fat diet-induced hepatosteatosis, α-naphthylisothiocyanate induced liver injury and diethylnitrosamine (DENA)-induced hepatocellular carcinoma El-Saied et al. (2018); Yang et al. (2012); Yang et al. (2016a), anti-hepatic fibrosis Jin et al. (2005)
	*Rheum offcinale* Baill					
	*Rheum tan*guticum Maxim.ex Balf					
2	*Angelica sinensis* (Oliv.) Diels	Radix Angelicae Sinensis	Apiaceae	Root	Blood deficiency and chlorosis^a^; Syndrome of blood deficiency^c^	Anti-inflammatory, anti-oxidative stress Mo et al. (2018)
3	*Silybum marianum* (L.)Gaertn	Herba Silybi	Asteraceae	Whole grass and achene	Fruit and extract for liver disease and jaundice^d^; Fatty liver, chronic hepatitis, cirrhosis^c^; Acute or chronic hepatitis, liver cirrhosis, fatty liver, metabolic toxic liver injury^b^	Protective effect on liver injury caused by cholestasis Alaca et al. (2017), protective effect against hepatotoxicity caused by deltamethrin Jindal et al. (2019), anti-oxidative stress Egresi et al. (2020); Zhu.et al. (2018a), regulating lipid metabolism Feng et al. (2019)
4	*Artemisia scoparia* Waldst. et Kit	Herba Artemisiae Scopariae	Asteraceae	Aboveground part	Infectious icteric hepatitis^a^ ^,^ ^d^	Anti-hepatocellular carcinoma Jang et al. (2017); Jung et al. (2018); Kim et al. (2018); Yan et al. (2018)
	*Artemisia capillaris* Thunb					
5	*Gentiana scabra *Bunge	Gentianae Radix et Rhizoma	Gentianaceae	Root and rhizome	Liver channel is hot and jaundice^d^; Damp-heat jaundice, head distension and headache caused by liver and gallbladder excess fire^c^	Anti-hepatic fibrosis Qu et al. (2015), protective effect on liver injury caused by B19-NS1 Sheu et al. (2017)
6	*Bupleurum chinense* DC.	Radix Bupleuri	Apiaceae	Root	Chest pain, irregular menstruation^a^ ^,^ ^c^ ^,^ ^d^	Protective effect on liver injury caused by acetaminophen and D-galactosamine/lipopolysaccharide Wang et al. (2019a); Zou et al. (2018), anti-oxidative, anti-inflammatory Jia et al. (2019), enhancing immune function Zou et al. (2019)
7	*Polygonum cuspidatum* Sieb. et Zucc	Rhizoma Polygoni Cuspidati	Polygonaceae	Root and rhizome	Damp-heat jaundice, amenorrhea in women^a^ ^,^ ^c^ ^,^ ^d^	regulating lipid metabolism, anti-oxidative stress, alleviating insulin resistance Kim. et al. (2020a); Zhao. et al. (2019a)
8	*Atractylodes macrocephala* Koidz	Rhizoma	Asteraceae	Rhizome	Jaundice^d^	Anti-acute liver injury Han et al. (2016)
		Atractylodis				
		Macrocephalae				
9	*Scutellaria baicalensis* Georgi	Radix	Labiatae	Root	Jaundice^a^; Headache due to liver fire, swelling and pain due to red eyes, damp-heat jaundice^c^	Relieving endoplasmic reticulum stress Dong et al. (2016), anti-hepatocellular carcinoma Wang. et al. (2020a), anti-oxidative stress, anti-inflammatory Park et al. (2017), anti-hepatic fibrosis Pan et al. (2015a)
		Scutellariae				
10	*Curcuma longa* L	Rhizoma Curcumae Longae	Zingiberaceae	Rhizome	Amenorrhea of women^a^; Women with blood stasis and amenorrhea^d^; Women have dysmenorrhea and amenorrhea^c^	Relieving endoplasmic reticulum stress Kim et al. (2017a), anti-oxidative stress, anti-inflammatory, protective effect on liver injury caused by CCl_4_, ethanol and methotrexate Lee. et al. (2017a); Moghadam et al. (2015); Uchio et al. (2017)
11	*Ligusticum chuanxiong* Hort	Rhizoma Chuanxiong	Apiaceae	Rhizome	Irregular menstruation, dysmenorrhea, chest pain^b^ ^,^ ^c^ ^,^ ^d^	Anti-hepatocellular carcinoma (Hu et al. 2015), protective effect against D-galactose-induced liver and kidney injury (Mo et al. 2017)
12	*Glycyrrhiza uralensis* Fisch	Radix	Leguminosae	Root	Hepatitis^b^	Hepatoprotective activities against CCl4/alcohol -induced liver injury Jung et al. (2016); Lin et al. (2017), anti-oxidative stress Cao et al. (2017)
	*Glycyrrhiza inflata* Bat	Glycyrrhizae				
	*Glycyrrhiza glabra* L					
13	*Prunus persica* (L.) Batsch	Semen Persicae	Rosaceae	Mature seed	Amenorrhea, dysmenorrhea^a^ ^,^ ^c^ ^,^ ^d^	Anti-hepatocellular carcinoma Shen et al. (2017), protective effect on liver injury caused by CCl_4_ Rehman et al. (2021), anti-oxidative stress, anti-inflammatory Kim. et al. (2017b); Lee et al. (2008)
	*Prunus davidiana* (Carr.) Franch					
14	*Sophora flavescens* Ait	Radix	Leguminosae	Root	Jaundice^a^ ^,^ ^c^ ^,^ ^d^	Anti-hepatitis B virus Yang et al. (2018)
		Sophorae Flavescentis				
15	*Sophora tonkinensis* Gapnep	Radix Sophorae Tonkinensis	Leguminosae	Root and rhizome	Jaundice^b^ ^,^ ^d^	regulating lipid metabolism, anti-oxidative stress, anti-inflammatory Zhao et al. (2020)
16	*Salvia miltiorrhiza* Bunge	Radix	Labiatae	Root and rhizome	Irregular menstruation, amenorrhea and dysmenorrhea^b^ ^,^ ^c^ ^,^ ^d^; Hepatosplenomegaly^b^	Protective effect on liver injury caused by paracetamol and lipopolysaccharide Gao et al. (2015); Zhou et al. (2015), anti-hepatocellular carcinoma Jiang et al. (2017b), anti-hepatic fibrosis Peng et al. (2018)
		Salviae Miltiorrhizae				
17	*Aloe barbadensis* Miller	Aloe	Liliaceae	The liquid concentrate of plant leaves	Liver heat^a^; Liver fire, headache, red eyes, convulsion^c^; Liver meridian excess heat, dizziness, headache, tinnitus, irritability, constipation^b^	Hepatoprotective effect against cartap- and malathion induced toxicityGupta et al. (2019), anti-inflammatory and anti-oxidant Klaikeaw et al. (2020)
	*Aloe ferox* Miller					
18	*Coptis chinensis* Franch	Rhizoma Coptidis	Ranunculaceae	Rhizome	Liver fire, red eyes, jaundice, disharmony between liver and stomach^a^; Liver fire, red eyes, swelling and pain^c^	Anti-hepatocellular carcinoma Auyeung and Ko. (2009); Lin et al. (2004); Ma et al. (2018a), anti-hepatitis C virus Hung et al. (2018), protective effect on liver injury caused by CCl_4_ Ma. et al. (2018b)
	*Coptis deltoidea* C. Y. Cheng et Hsiao					
	*Coptis teeta* Wall					
19	*Paeonia lactiflora* Pall	Radix	Ranunculaceae	Root	Hypochondriac pain, blood deficiency and chlorosis, Irregular menstruation^a^; Chest and abdomen rib pain, irregular menstruation^b^ ^,^ ^d^	Improving liver function, anti-inflammatory and anti-oxidant Wang. et al. (2020b)
		Paeoniae Alba				
20	*Paeonia lactiflora* Pall	Radix Paeoniae Rubra	Ranunculaceae	Root	Eye red swelling and pain, liver depression, hypochondriac pain, amenorrhea and dysmenorrhea^a^ ^,^ ^c^	Protective effect on liver injury caused by cholestasis Ma et al. (2018a); Ma et al. (2015)
	*Paeonia veitchii* Lynch					
21	*Isatis indigotica* Fort	Folium Isatidis	Brassicaceae	leaf	Jaundice^a^ ^,^ ^c^; Jaundice, acute infectious hepatitis^d^; Acute hepatitis^b^	Enhancing the endogenous antioxidant system Ding and Zhu (2020)
22	*Isatis indigotica* Fort	Radix Isatidis	Brassicaceae	Root	Acute and chronic hepatitis^d^; Hepatitis^c^	Alleviating insulin resistance Li et al. (2019b)
23	*Lycium barbarum* L	Fructus Lycii	Solanaceae	fruit	The eyes are not clear^a^; Yin deficiency of liver and kidney, dizziness^c^ ^,^ ^d^	Protective effect against paracetamol-induced acute hepatotoxicity Gündüz et al. (2015), anti-hepatocellular carcinoma Ceccarini et al. (2016), Regulating the immune system Tan et al. (2019)

a Cited from “Chinese Pharmacopoeia.”

b Cited from “Zhong Yao Da Ci Dian”.

c Cited from “Zhong Hua Ben Cao”.

d Cited from “Quan Guo Zhong Cao Yao Hui Bian”.

(Note: doctor of traditional Chinese medicine holds that the liver stores blood and the liver is a sea of blood).

### Regulating Lipid Metabolism

Lipid uptake, esterification, oxidation, and fatty acid secretion all occur in hepatocytes. These processes are regulated by hormones, nuclear receptors, and transcription factors to maintain liver lipid homeostasis (Nguyen et al., 2008). If the balance of liver lipid metabolism is destroyed, the lipid will accumulate abnormally in the liver. Excessive lipid accumulation will lead to liver steatosis, insulin resistance and the development of fatty liver disease, and even induce oxidative stress, causing inflammation, cytotoxicity and aggravating liver injury. Therefore, maintaining normal lipid metabolism is an important function of the liver (Ding, H.-R. et al., 2018; Li, X. et al., 2019).

Many CMs have shown good effects in regulating lipid metabolism, such as *Radix Bupleuri*, *Pericarpium Citri Reticulatae*, *Rhubarb*, *Polygonum Multiflorum*, *Coptis Chinensis*, *Artemisia Annua*, *Flos Lonicera* and *Radix Sophorae Tonkinensis*. The results showed that the serum high-density lipoprotein cholesterol (HDL-C), TC and low-density lipoprotein cholesterol (LDL-C) levels of c57BL/6 mice were reduced by Citrus reticulata Blanco peel extract. The author further revealed that 0.2 and 0.5% of the extract could effectively prevent the micro fatty degeneration and excessive accumulation of lipid droplets in the liver (Ke et al., 2020). *Rheum Palmatum* L. can continuously reduce the accumulation of excess fat and the expression of lipogenic genes in the liver of male Sprague-Dawley rats induced by a high-fat diet. Concomitantly, increased phosphorylation of adenine monophosphate activated protein kinase (AMPK) and acetyl-CoA carboxylaze was observed (Yang, M. et al., 2016). In addition, *Sophorae Tonkinensis* water extract and *Polygonum Multiflorum* Thunb. extract alleviate nonalcoholic liver disease by enhancing hepatic carnitine palmitoyltransferase 1A activity to promote fatty acids β-oxidation, and regulating the protein response to lipid metabolism and expression in the liver to reduce lipid accumulation (Jung et al., 2020; Zhao et al., 2020).

It is worth mentioning that relevant studies of hepatic lipid metabolism were also conducted in fish. Addition of 200–400 mg/kg *Radix Bupleuri* extract to the daily diet of hybrid grouper fish can reduce the expression of lipogenesis-related genes, such as diacylgycerol acyltransferase 2, glucose-6-phosphate dehydrogenase, malic enzyme 1 and diacylglycerol kinase alpha (Zou et al., 2019). Lonicera japonica extract can effectively reduce the levels of LDL-C, triglyceride (TG) and total cholesterol (TC) in the serum of grass carp as well as the expression of lipogenic genes acc1, fas, SREBP1 and PPARγ, and increase the expression of liposoluble genes CPT1, ATGL, LPL and PPARα (Meng et al., 2019).

### Liver Injury

#### Liver Fibrosis

Liver fibrosis belongs to chronic liver injury, mainly manifested as the accumulation of extracellular matrix (Tsuchida and Friedman, 2017), which is a dynamic process. Hepatocytes, activated hepatic stellate cells, endothelial cells, immune cells, and macrophages all participate in its establishment and regression (Campana and Iredale, 2017). Liver fibrosis is a pathological insult mainly caused by chronic liver disease (viral infection, alcoholic liver disease, NASH, etc). If not treated in time, it will continue to deteriorate and eventually progress to cirrhosis and even liver cancer.

The TGF-β/Smads pathway plays an important role in the regulation of liver fibrosis. In the background of liver fibrosis, Smad3 and Smad4 are pro-fibrosis, while Smad2 and Smad7 are anti-fibrosis (Xu et al., 2016). Meanwhile, TGF-β is also activated by the deposits in the fibrous extracellular matrix, and expressed and released from a variety of cells (Dewidar et al., 2019). The evidence has shown that *Forsythiae Fructuse* water extract (FSE), *Curcuma Wenyujin*, and *Zingiber Officinale* can effectively inhibit the development of liver fibrosis through the TGF-β/Smads signaling pathway (Hasan et al., 2016; Hu et al., 2020a; Xie et al., 2020).

*Radix Salvia Miltiorrhiza* (RSM) is the dry root and rhizome of Labiatae plant *Salvia Miltiorrhiza* Bunge, whose main functions include removing blood stasis, relieving pain, activating blood circulation, clearing the heart, and removing trouble (Commission, 2015). It is widely used in the treatment of liver fibrosis in clinic, but the specific mechanisms are not clear. The recent study of Yuan et al. showed that RSM improved liver fibrosis by increasing the activity of natural killer (NK) cells as well as the effects of NKG2D and NKp46 on NK cells, and inhibiting the activation of HSCs *in vivo* and *in vitro* (Peng et al., 2018). Another study showed that the mixture of RSM extract and *Astragalus Membranaceus* extract at a ratio of 1:1 could regulate the expression of TGF-β1 and Cyclin D1 to improve liver fibrosis and the liver functions, especially having a good effect on reducing the cyclin D1 expression (Cao et al., 2020). In addition, many CM have anti-fibrosis activities. For example, *Gentiana Scabra* bage inhibits fibrosis by reducing the expression of hepatic type I and type III collagen proteins in rats (Qu et al., 2015). *Ginkgo biloba* is also a common CM mainly used in coronary heart disease, angina pectoris, and hyperlipidemia (Commission, 2015). Wang et al. found that *Ginkgo bilob*a extract could improve liver fibrosis by inhibiting inflammation, HSC activation, and hepatocyte apoptosis, which may be related to the p38MAPK, NF-κB/IκBα, and Bcl-2/Bax signaling pathway (Wang et al., 2015).

#### Chemical Liver Injury

Chemical liver injury is mainly caused by alcohol, toxic chemicals, and drugs. As we all know, the liver has dual blood supply of hepatic artery and hepatic vein, which is the main detoxification organ of human body. The liver plays a core role in biotransformation and excretion of foreign compounds, so it is the main target of the adverse reactions of drugs and other heterologous organisms (Holt and Ju, 2010). Secondly, the liver is the initial contact site of alcohol, chemical toxic substances, and the oral drugs absorbed through the intestine, so it is vulnerable to chemical damage. At the same time, electrophilic compounds and free radicals are the intermediate products of many chemical substances after liver metabolism. These substances may change the structure and function of cell macromolecules, and even lead to the occurrence of liver cancer (Gu and Manautou, 2012).

At present, a variety of CM are widely used for chemical injuries. Both *Schisandra Sphenanthera* extract and *Polygonatum Sibiricum* water extract can regulate alcoholic liver injury in mice through the nuclear factor-erythroid 2-related factor 2 (Nrf2)-antioxidant responsive element (ARE) signaling pathway (Wang, G. et al., 2021; Zeng et al., 2017). The liver damage caused by CCl4 can be alleviated by *Curcuma longa* L. extract and *Prunus persica* Seeds Extract, which is mainly related to inhibiting liver oxidative stress, and increasing the Nrf2 and NQO-1 levels, as well as reducing type Ⅲ collagen mRNA expression (Lee, G.-H. et al., 2017; Rehman et al., 2021). In addition, *Hedyotis Diffusa* water extract, *Ligusticum Chuanxiong* Hort, and *Panax ginseng* can also be used to respectively relieve the chemical damage caused by hydrogen peroxide and D-galactose (Gao et al., 2016; Mo et al., 2017). It is worth mentioning that a large number of CM can also alleviate drug-induced liver injuries. Paracetamol (acetaminophen) is a commonly used drug in clinic, which is mainly used for cold-induced fever, headache, joint pain, neuralgia, migraine, dysmenorrhea, and so on. *Lycium Barbarum* extract can significantly improve paracetamol-induced apoptosis to protect the liver from chemical damage (Gündüz et al., 2015), and *Isatidis Folium* can enhance the endogenous antioxidant system and reduce paracetamol-induced liver damage in mice (Ding and Zhu, 2020). Ahmed et al. also found that *Panax ginseng* could be used as a hepatoprotective agent, which prevented cyclophosphamide (with immunosuppressive and anti-cancer potential)-induced liver injury by reducing the expression of TNF-α, IL-1β and Caspase3 genes, as well as increasing the BCL-2 gene expression, and its liver-protective effect is better than vitamin E (Abdelfattah-Hassan et al., 2019).

#### Anti-oxidative Stress

Oxidative stress is the main influencing factor of the pathogenesis of ALD and MAFLD. It has been briefly discussed in the previous content. When the level or activity of antioxidants in the human body is reduced, oxidative stress will occur. Due to the stimulation of external factors (such as alcohol), the body will produce a large amount of active oxygen, which is the key to the development of fatty liver into steatohepatitis. GSH is an endogenous antioxidant, which is widely present in animals. Excessive oxidative stress can cause GSH consumption and lead to the accumulation of ROS (Li, X. et al., 2019). In addition, cytochrome P4502E1 (CYP2E1) plays a key role in the generation of ROS, which is also induced by alcohol (Leung and Nieto, 2013). *Calculus bovis* is a commonly used CM for fever, faintness, stroke and phlegm. The evidence showed that *calculus bovis* could inhibit oxidative stress in hepatocytes by reducing ROS and increasing SOD content, thereby achieving the liver-protective effect on mice with nonalcoholic fatty liver. And *curcuma longa* hot water extract and *zingiber officinale* hydroalcoholic extract can reduce the level of GSH to protect the liver.

**FIGURE 4:**
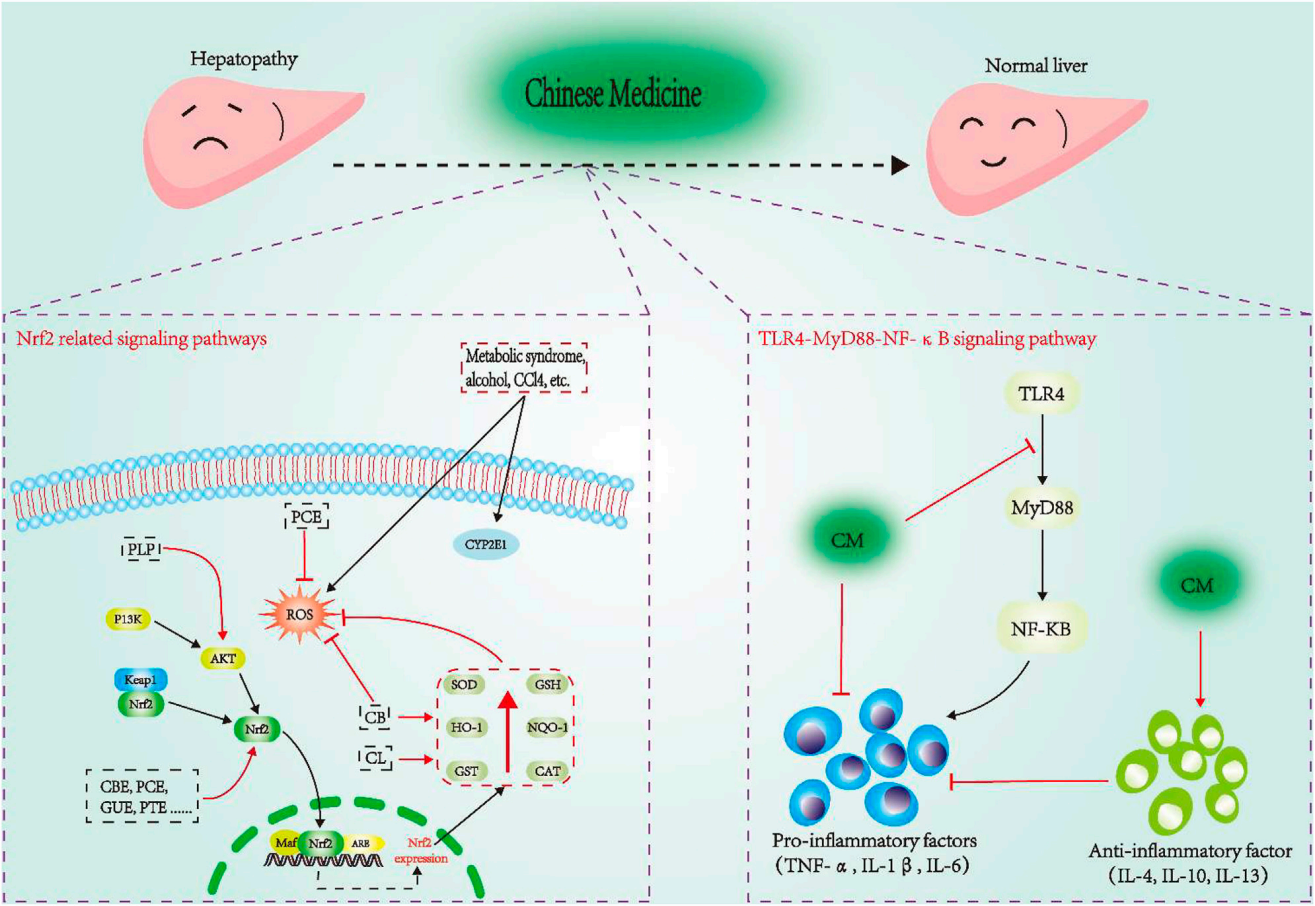
Some CM treat liver disease through Nrf2 and TLR4-MyD88-NF-κB signaling pathway. CBE, Citrus reticulata Blanco peel extract; GUE, Glycyrrhiza uralensis ethanol extract; PTE, Polygonum multiflorum Thunb. ethanolic extract; PLP, Paeonia lactiflflora Pall.; CL, Curcuma longa; CB, Calculus bovis; PCE, Polygonum cuspidatum extract.

Nrf2 is an important redox-sensitive transcription factor, and controls the basic and induced expression of a series of antioxidant response element-dependent genes, which is beneficial to improve the body’s oxidative stress state, thus regulating the physiological and pathological consequences under oxidant exposure (Ma, 2013). Under normal physiological conditions, Nrf2 is locked in the cytoplasm by Keap1. But when the cells are attacked by ROS or electrophiles, Nrf2 will dissociate from Keap1 and quickly translocate into the nucleus, first forming a heterodimer with the small Maf protein, and then combining with the ARE, which finally transcribes and activates the expression of the antioxidant enzyme genes regulated by Nrf2 (Ho et al., 2012; Heiss et al., 2013). In addition, the signal pathways related to Nrf2 (such as Nrf2-Keap1 and Nrf2-ARE) in the oxidative stress system have been widely recognized, especially the Nrf2-Keap1 pathway, which is an anti-stress mechanism inherited from our ancestors, as well as a defense system to maintain the homeostasis of the cells (Buendia et al., 2016; Bellezza et al., 2018). As reported, *Polygonum Cuspidatum* extract could reduce oxidative stress by targeting the Keap1/Nrf2 pathway, and down-regulate the levels of sterol regulatory element bending protein 1, fatty acid synthase, and stearoyl coenzyme alpha desaturase-1 to prevent hepatic lipid accumulation in fructose-fed rats (Zhao, X.-J. et al., 2019). *Paeonia Lactiflora* Pall. (PLP) can increase the expression of AKt, Nrf2, HO-1, NQO1 and GCLC, and activate the PI3K/Akt/Nrf2 pathway to enhance the antioxidant system, thereby reducing ANIT-induced liver tissue damage (Ma et al., 2015). In addition, *Citrus Reticulata* Blanco peel extract, Glycyrrhiza Uralensis ethanol extract, and *Polygonum Multiflorum* Thunb. ethanolic extract can directly activate the Nrf2 to regulate the redox state of liver injury (Cao et al., 2017; Ke et al., 2020; Lin, E.-Y. et al., 2018). The details are showed in Figure 4.

## Regulation of Bile Acid Metabolism

Bile acids (BAs) are important components of bile, which have the functions of regulating metabolism, endocrine and immune (Chávez-Talavera et al., 2017). The liver is the site of bile acid synthesis. The primary bile acids, such as cholic acid and chenodeoxycholic acid, combine with glycine or taurine to form bound BAs, which are secreted into bile canaliculus through the transport proteins such as bile salt export pump and multidrug resistance associated protein 2, and are temporarily stored in the gallbladder and released through the bile duct. When BAs and other components of bile are discharged into the intestine together, they can promote the emulsification and absorption of dietary fat, cholesterol, and fat-soluble vitamins. About 90–95% of BAs are reabsorbed in the ileum through apical sodium-dependent bile acid transporter and ileal bile acid transporter (IBAT), and the remaining 5–10% of BAs are excreted in feces (Li and Chiang, 2014; Tripathi et al., 2018). BAs are the important physiological basis involved in the regulation of liver function and disease states. According to the data, the metabolism and inflammation related to obesity, type 2 diabetes, dyslipidemia, and MAFLD are all regulated by BAs (Chávez-Talavera et al., 2017). Therefore, BAs’ normal synthesis, transportation and excretion are vital factors for the homeostasis.

Cholestasis means that the bile cannot flow from the liver to the duodenum, and its flow is decreased, which is characterized by the excessive accumulation of bile acids and other toxic compounds (Crocenzi et al., 2012). Excessive accumulation of bile acids in the liver may cause liver damage, liver fibrosis, and eventually liver failure and biliary cirrhosis (Padda et al., 2011). The study has shown that PLP can regulate glycocholic acid, taurocholic acid, glycodeoxycholic acid, L (D)-arginine, and L-tryptophan, and these metabolites are related to bile acid secretion and amino acid metabolism, which is concluded that bile acid metabolism may be involved in the therapeutic effects of PLP on cholestasis (Ma et al., 2016). Ma et al. further demonstrated that PLP could alleviate cholestasis by regulating the NF-κB-NLRP3 inflammasome and the PI3K/Akt-dependent pathways (Ma, X. et al., 2018; Ma et al., 2015). Another study showed that the ethanol extract of *Schisandra Chinensis* could significantly protect the mice from intrahepatic cholestasis induced by cholic acid (Zeng et al., 2016). In addition, *Schisandra Chinensis* extract can also enhance the excretion of bile acids from the serum and liver to the intestine and feces, and adjust the intestinal microorganisms disturbed by the external factors to achieve the protective effects on liver injury caused by cholestasis (Li, D.-S. et al., 2020).

## Regulating the Immune System

### Inhibition of Inflammatory Response

Inflammation is the basis of a variety of physiological and pathological processes, mainly induced by infection and tissue damage (Medzhitov, 2008). When natural antioxidants are out of balance, the free radicals produced by different organisms and environments can further lead to various inflammation-related diseases (Arulselvan et al., 2016). As we all know, there are many kinds of cytokines involved in the inflammatory response. For example, TNF-α, IL-1β, and IL-6 play a pro-inflammatory role, by contrary, TGF-β, IL-4, IL-10, and IL-13 can inhibit the occurrence and progress of inflammation. There is evidence that the inflammatory mechanisms of the liver are essential for maintaining the homeostasis of the tissues and organs. When the inflammatory mechanisms are out of balance, the hepatic pathological process will be drived, such as chronic infection, autoimmunity, and malignant tumor (Robinson et al., 2016). FSE, *Gentianae Macrophyllae* extract, and *Aloe vera* can reduce inflammatory liver injury by reducing the serum concentration of TNF-α, IL-1β, IL-6, NF-κB, and other cytokines (Zhao et al., 2017; Cui et al., 2019; Hu et al., 2020a; Klaikeaw et al., 2020). Moreover, *Radix Bupleuri* extract and *Schisandra Sphenanthera* extract can directly inhibit the mRNA expression of TNF-α, IL-1β, and IL-6 to protect the liver (Chen et al., 2019; Jia et al., 2019). In addition, *Angelica Sinensis* Supercritical Fluid CO2 Extract can significantly inhibit D-galactose-mediated expression of inflammatory cytokines, such as iNOS, COX-2, IKBα, *p*-IκBα, and p65, protecting the liver and kidney tissues (Mo et al., 2018).

Toll-like receptor4 (TLR4)-myeloid differentiation factor 88 (MyD88)-NF-κB signaling pathway is a key pathway in the physiologic and biochemical reactions of diseases. It widely exists in various tissues and cells, which is one of the important signaling pathways that mediate the expression of inflammatory factors (Wu et al., 2017). As one of the important pathways associated with inflammatory response and hepatic fibrosis, its activation can lead to the release of downstream inflammatory factors and induce the production of TNF-α, IL-1β, and IL-6. Hu et al. found that FSE could improve the inflammatory state of liver fibrosis through the TLR4-MyD88-NF-κB pathway (Hu et al., 2020a). Jia et al. found that RBE could inhibit TLR4-MyD88-NF-κB signaling pathway to reduce H_2_O_2_-induced liver inflammation in tilapia (Jia et al., 2019). Another study showed that GME could also attenuate ALD by inhibiting the phosphorylation of JNK and p38 to inhibit the initiation of inflammation (Cui et al., 2019).

The molecular mechanisms of the CM alleviating liver diseases through inflammatory pathways are shown in Figure 4.

### Enhancing Immune Function

Zou et al. found that adding 200–800 mg/kg RBE to the diet of hybrid grouper could effectively reduce the serum ALP, ALT, AST, and LDH contents. In addition, it could down-regulate the expression of apoptosis-related genes (caspase-9), and up-regulate the antioxidant genes (CAT) and immune-related genes (MHC2, IKKα, and TGF-β1) (Zou et al., 2019). Tan et al. reported that dietary supplementation of *Lycium barbarum* extract (0.50–2.00 g/kg) could effectively increase IL-10 and TGF-β1 mRNA levels in the liver of HFD-fed hybrid grouper (Tan et al., 2019). In addition, *Ginkgo biloba* extract not only improves the hepatic antioxidant status of HFD-fed hybrid grouper, and maintains normal liver histology and preserves liver function, but also up-regulates the expression of immune-related genes (MHC2 and TLR3) (Tan et al., 2018).

### Hepatitis Virus

Some CM have inhibitory effects on hepatitis virus and can assist the treatment of patients with viral hepatitis. Some studies have shown that most of the terpenoids isolated from *Flos Lonicerae* can inhibit the secretion of HBsAg and HBeAg, as well as the DNA replication of HBV (Ge et al., 2019). In addition, Yang et al. found that the methanolic extract of *Rhizoma Coptidis* could block the attachment of HCV and the entry/fusion with host cells, which effectively inhibited the infection of pseudoparticles of HCV in Huh-7.5 cells, and hindered the infection of several HCV genotypes (Hung et al., 2018).

### Liver Cancer

Currently, Western medicine and therapies are the main treatment strategies for liver cancer, but the overall prognosis of liver cancer patients is still very poor. Under such circumstances, it is extremely urgent to find a better method for the treatment of liver cancer. CM contains abundant treatment resources and has been used for the prevention of liver cancer for thousands of years. In modern China, CM has also been proven to be an effective method for the treatment of liver cancer. However, the theory of CM prevention and treatment of liver cancer is more widely accepted in China than abroad (Liao et al., 2020). According to relevant data, most CM can show anti-liver cancer effects. Ethanol extract of root of *Prunus Persica* can significantly inhibit the migration of liver cancer HepG2 cells and the expression of extracellular matrix metalloproteinases, MMP3 and MMP9. It is worth mentioning that it can also inhibit tumor growth in nude mice *in vivo* (Shen et al., 2017). *Artemisia capillaris* extract can inhibit the growth, migration and invasion of Huh7 and HepG2 liver cancer cells. This inhibitory effect is closely related to blocking the PI3K/AKT signaling pathway (Yan, Honghua et al., 2018). Jiang et al. further found that the anti-liver cancer effect of *Artemisia capillaris* extract is also related to the inhibition of the IL-6/STAT3 signal axis (Jang et al., 2017). Futhermore, Zheng et al. found that oral administration of *portulaca oleracea* extract to male AKR mice for seven consecutive days could contribute to the treatment of liver cancer. The results showed that the serum levels of IL-6, IL-1β, TNF-α and MDA in mice decreased after 7 days of treatment, while the activity of SOD increased. The pathological changes of the liver were significantly alleviated. Meanwhile, *portulaca oleracea* extract could effectively inhibit PI3K, Akt, mTOR, NF-κB and IκBα, and up regulate the expression of Nrf2 and HO-1. These effects are attributed to the protective effect of *Portulaca oleracea* extract on liver cancer by regulating PI3K/Akt/mTOR and Nrf2/HO-1/NF-κB pathway (Guoyin et al., 2017).

In addition, some CM can also achieve protection against liver cancer through various other effects. For examples, *Astragalus membranaceus* and *Curcuma wenyujin* promote the normalization of blood vessels in liver tumor endothelial cells by increasing the expression of CD34 and reducing the expression of HIF1a (Zang et al., 2019). *Artemisia capillaris* leaves can achieve pro-apoptotic effects on liver cancer cells by reducing the expression of XIAP and the release of cytochrome C through mitochondrial membrane potential (Kim et al., 2018). Besides, *Ligustrum lucidum* Ait. fruit extract can induce apoptosis and cell senescence of human liver cancer cell Bel-7402 by up-regulating p21. All in all, there are abundant resources of CM against liver cancer, which are worthy of our further development and utilization.

### Other Anti-liver Disease Mechanisms

A large number of studies have shown that the occurrence of liver diseases is also closely related to endoplasmic reticulum stress and insulin resistance. *Scutellaria baicalensis* Georgi extract can regulate the endoplasmic reticulum stress and protect the liver by reducing the expression of glucose-related protein 78 (Dong et al., 2016). HFD increased the expression of adipose-derived carbohydrate response element binding protein and endoplasmic reticulum stress genes CHOP, x-box binding protein 1, and glucose regulated protein 78 in male wistar rats, and *Ginger* extract could restore these changes to normal state (Kandeil et al., 2019). Jung et al. reported that *Polygonum multiflfluorum* thunb. reduced nonalcoholic steatosis and insulin resistance by regulating the expression of the proteins on lipid metabolism and glucose transport in the liver (Jung et al., 2020).

Recently, the evidence has shown that gut microbiota play an important role in metabolism, immune system, and so on. The changes of gut microbiota and their function can promote the development of acute and chronic liver diseases. In addition, the destruction of intestinal barrier can make microorganisms transfer to the blood, and continuously cause inflammatory reaction, thus promoting liver injury, hepatic fibrosis, cirrhosis, and carcinogenic transformation (Shen et al., 2018; Chopyk and Grakoui, 2020). *Rhubarb* extract can promote some intestinal bacteria (such as *Akkermansia muciniphila* and *Parabacteroides goldsteinii*.) to participate in the intestinal barrier function, and alleviate liver inflammation caused by acute alcohol intake (Neyrinck et al., 2017). In addition, *Schisandra chinensis* bee pollen could inhibit the expression of LXR-α, SREBP-1c, and FAS genes, and regulate the structure of intestinal microflora in obese mice, so as to achieve the protective effect on MAFLD (Cheng et al., 2019).

## Natural Agents From CM for Liver Disease Treatment

### Polysaccharides and Glycosides

Polysaccharide is one of the active components of CM. The polysaccharides in CM have a wide range of biological activities in enhancing immunity, antiviral, anti-inflammation, anti-oxidation, and anti-tumor (Chen et al., 2016). *Ginkgo biloba* leaf polysaccharides and *Astragalus* polysaccharides can effectively inhibit liver steatosis (Yan et al., 2015; Huang et al., 2017). The polysaccharides from roots of *Sophora flavescens* can significantly inhibit the HBsAg and HBeAg secretion of HepG2.2.15 cells, and have good anti-HBV activity (Yang et al., 2018). In addition, the polysaccharides extracted from many CM have obvious protective effects on acute liver injury, such as *Rhizoma Atractylodis Macrocephalae* polysaccharides (Han et al., 2016), *Angelica sinensis* polysaccharides (Wang, K. et al., 2020), *Poria Cocos* polysaccharides (Wu, K. et al., 2018), *Lycium barbarum* polysaccharides (Wei et al., 2020), and *Schizandra chinensis* acidic polysaccharides (Yuan et al., 2018). Wang et al. reported that *Paeoniae Radix Alba* polysaccharides inhibited the NF-κB signaling pathway (including the liver infiltration of inflammatory CD^4+^ and CD^8+^ cells, and the overexpression of inflammatory cytokines IL-2, IL-6, and IL-10) to inhibit the immune inflammatory response in experimental autoimmune hepatitis mice (Wang, S. et al., 2020). Finally, it is also important that APS is the main active component extracted from *Astragalus*, which has been proved to have a significant inhibitory effect on many types of human solid tumors. A recent study showed that APS could reduce the activity of hepatoma cells and induce the apoptosis of HCC cells in a concentration-dependent manner. The study further showed that the results might be related to inhibiting the expression of Notch 1 in HCC cells (Huang et al., 2016).

Glycosides are a class of compounds formed by linking the sugar or sugar derivative with another non-sugar substance through the terminal carbon atom of the sugar. The studies have shown that most glycosides have good hepatoprotective effects on liver, such as amygdalin, amarogentin, and forsythiaside A (Pan, C.-W. et al., 2015; Tang et al., 2019; Zhang et al., 2017). Chrysophanol 8-o-glucoside, extracted from *Rheum palmatum*, can significantly inhibit the gene expression of α-SMA and collagen I, and inhibit the phosphorylation of STAT3 by inhibiting the nuclear translocation of p-STAT3, thus alleviating fibrosis and achieving liver protection (Park et al., 2020). What’s more, Gentiopicroside not only protects alcoholic liver disease by improving lipid metabolism imbalance and mitochondrial dysfunction caused by alcohol (Yang, H.-X. et al., 2020; Zhang et al., 2021), but also treats alcoholic liver cancer by regulating the activation of P2x7R-NLRP3 inflammasome (Li, Xia et al., 2018). It is worth mentioning that astragaloside IV can inhibit hepatoma cells by inhibiting multidrug resistance-associated protein 2, and long noncoding RNA ATB (Li, Y. et al., 2018; Qu et al., 2020).

**FIGURE 5:**
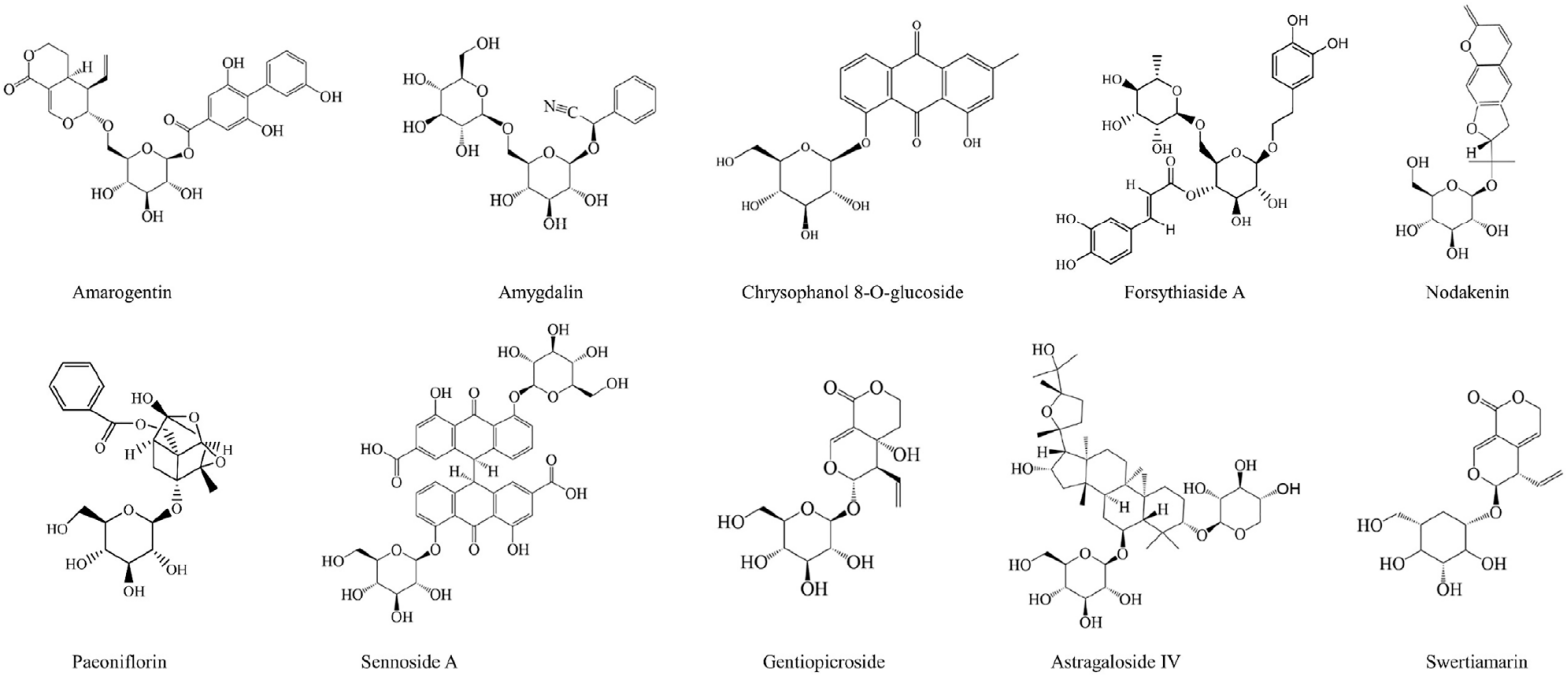
The chemical structures of glycosides showing anti-hepatopathy activity.

The specific information of polysaccharides and glycosides is shown in Table 2. In addition, the chemical structures of the glycosides with therapeutic effects on liver diseases are shown in Figure 5.

**TABLE 2:** Summary of polysaccharides and glycosides with significant anti-liver disease activity.

Compounds	Source	The species investigated	Dose	Mechanisms	References
Polysaccharides					
PRAM2	*Rhizoma Atractylodis Macrocephalae*	Male ICR mice	50, 100, 200 mg/kg	Inhibition of NOS activity and NO level and its reduction of the production of free radicals	Han et al. (2016)
Radix isatidis polysaccharide	*Radix isatidis*	3T3-L1 preadipocytes	25, 50, 100 μg/ml	Improvement of the glucose metabolism, lipid metabolism and oxidative stress	Li, et al. (2019c)
		Male Wistar rats	25, 50, 100 mg/kg		
Salvia miltiorrhiza polysaccharide	*Salvia miltiorrhiza*	Chickens	0.5, 1, 2 g/L	Down-regulation of the contents of ALT, AST, and MDA, and up-regulation of the contents of GSH and CYP450	Han et al. (2019)
		Chicken hepatocytes	100, 200, 500 μg/ml		
Angelica sinensis polysaccharide	*Angelica sinensis*	L02 cells	200, 400, 800 μg/ml	Through regulating lipid metabolism, anti-inflammation, anti-oxidation and inhibiting HSC activation	Ma et al. (2020); Wang et al. (2016); Wang. et al. (2020c)
		ICR male mice	100, 300, 500 mg/kg		
		Male Balb/c mice	1.5, 6 mg/kg		
		Murine splenocytes	5, 25,125 μg/ml		
		Male C57BL/6J mice	200 mg/kg		
		Primary splenocytes	50, 100, 200 μg/ml		
Codonopsis pilosula polysaccharide	*Codonopsis pilosula*	Female ICR mice	100, 150, 200 mg/kg	Through antioxidant effect	Liu et al. (2015)
Poria cocos polysaccharide	*Poria cocos*	Male Kunming mice	200, 400 mg/kg	By suppressing cell death, reducing hepatocellular inflammatory stress and apoptosis, and Hsp90 bioactivity	Wu et al. (2018c); Wu. et al. (2019b)
		AML12 cells	20, 40 g/L		
Lycium barbarum polysaccharide	*Lycium barbarum*	L02 cells	24 μg/ml	By reversing oxidative injury, inflammatory response and TLRs/NF-κB signaling pathway expression	Gan. et al. (2018b); Wei et al. (2020)
		Male wistar rats	400, 800, 1600 mg/kg		
Astragalus membranaceus-Polysaccharide	*Astragalus membranaceus*	HFSTZ Mice	500 mg/kg	Through improving peripheral metabolic stress, activating hepatic insulin signaling	Huang et al. (2016); Huang et al. (2017); Sun et al. (2019)
		C57BL/6 mice	800 mg/kg		
		HCC cells	0.1, 0.5, 1 mg/ml		
SFP-100	*Sophora flavescens*	Female Balb/c mice	500 mg/kg	By decreasing hepatocytes apoptosis, inhibit the infiltration of neutrophils and macrophages into liver	Yang et al., (2018)
		L02 cells	10, 50, 250 μg/ml		
		HepG2.2.15 cells	50, 100, 250, 500 μg/ml		
Codonopsis lanceolata polysaccharide	*Codonopsis lanceolata*	Male C57BL/6 mice	100 mg/kg	Through activating anti-oxidative signaling pathway	Zhang, et al. (2020a)
STRP	*Sophora tonkinensis*	Male ICR mice	50, 100, 200 mg/kg	By inhibiting MDA, ROS generation and increasing liver GSH, GPx, T-SOD, CAT levels	Cai et al. (2018); Shan et al. (2019)
Schisandra chinensis Polysaccharide	*Schisandra chinensis*	Mice	200, 400, 800 mg/kg	Regulation of Nrf2/antioxidant response element and TLR4/NF-κB signaling pathways	Shan et al. (2019)
Schisandra chinensis acidic polysaccharide	*Schisandra chinensis*	Male ICR mice	5, 10, 20 mg/kg	By inhibiting the expression of CYP2E1 protein and then alleviating oxidative stress injury	Yuan et al. (2018)
		HepG2 cells	3.12, 6.25, 12.5 μg/ml		
GBLP	*Ginkgo biloba*	Male Wistar rats	100, 200, 400 mg/kg	By attenuating IR, preserving liver function, enhancing antioxidant defense system, and reducing lipid peroxidation	Yan et al. (2015)
Paeoniae radix alba polysaccharides	*Paeoniae radix alba*	Male Kunming mice	0.2, 0.4, 0.8 g/kg	Inhibition of the NF-κB signaling pathway	Wang et al. (2020b)
Glycosides					
Chrysophanol 8-O-glucoside	*Rheum palmatum*	LX-2 cells	1, 5, 20 μg/ml	Regulation of the STAT3 signaling pathway	Park et al. (2020)
Sennoside A	*Rheum officinale Baill*	HepG2 cells	25, 50, 100 μM	Down-regulation of KRT7 and KRT81, and inhibition of the AKT and ERK pathways	Le et al. (2020); Zhu et al. (2020)
		SMMC-7721 cells	25, 50, 100 μM		
		Male C57BL/6J mice	15, 30, 60 mg/kg		
		HSC-T6 cells	10 μM		
Astragaloside IV	*Astragalus membranaceus*	SMMC-7721 cells	80 μg/ml	Inhibition of lncRNA-ATB, MRP2, PTP1B and anti-apoptotic signaling, and improvement insulin resistance	Li et al. (2018g); Qu et al. (2020); Su et al. (2020); Zhou et al. (2021)
		Huh-7 cells	80 μg/ml		
		HepG2 cells	0.4, 4, 40 μM		
		H22 cells	0.4, 4, 40 μM		
		Male BALB/c mice	50 mg/kg		
		HepG2 cells	6.4, 12.8, 25.6, 51.2, 102.4 μM		
		SK-Hep1 cells	200, 400 μM		
		Hep3B cells	200, 400 μM		
Amarogentin	*Swertia* and *Gentiana* roots	HSCs	0.01, 0.1, 1 mg/ml	By anti-oxidative properties and suppressing the mitogen-activated protein kinase signaling pathway	Zhang et al. (2017)
		Male C57BL/6 mice	25, 50, 100 mg/kg		
Amygdalin	*Armeniaca semen*	Female BALB/c mice	4, 8 mg/kg	regulation of the NLRP3, NF-κB, Nrf2/NQO1, PI3K/AKT and JAK2/STAT3 signaling pathways	Tang et al. (2019); Wang et al. (2021a); Yang et al. (2019a)
		HepG2 cells	80 μM		
		Male Sprague–Dawley rats	0.5, 1, 1.5, 3 mg/kg		
		LX-2 cells	1.25, 2.5, 5 mg/ml		
Forsythiaside A	*Forsythia suspensa*	Male BALB/c mice	15, 30, 60 mg/kg	Through modulating the remolding of extracellular matrix, PI3K/AKT and Nrf2 signaling pathway, and inhibition of NF-κB activation	Gong et al. (2021); Pan, et al. (2015a)
		Transgenic zebrafish	25, 50, 100 μM		
Gentiopicroside	*Gentiana manshurica Kitagawa*	Male Sprague–Dawley rats	20 mg/kg	Improvement of mitochondrial dysfunction and activation of LKB1/AMPK signaling	Li, et al. (2018e); Yang et al. (2020a); Zhang et al. (2021)
		Male C57BL/6 mice	40, 80 mg/kg		
		HepG2 cells	100 μM		
		RAW 264.7 macrophages	25, 50, 100 μM		
Paeoniflorin	*Paeonia lactiflora*	Male Sprague-Dawley rats	10, 20, 40, 80, 200 mg/kg	By activating LKB1/AMPK and AKT pathways, and inhibiting HMGB1-TLR4 signaling pathway and HIF-1α expression	Li, et al. (2018d); Xie et al. (2018); Zhao et al. (2014)
		Male C57BL/6 mice	100 mg/kg		
Swertiamarin	*Gentiana manshurica* Kitag	HSCs cells	2.4, 6, 15 μM	By suppressing angiotensin II–angiotensin type 1 receptor–extracellular signal-regulated kinase signaling	Li et al., (2016)
		MaleWistar rats	15, 20 mg/kg		
Nodakenin	*Angelica biserrata*	Male ICR mice	10, 30 mg/kg	By regulating apoptosis-related mitochondrial proteins	Lim et al. (2021)
Geniposide	*Gardenia jasminoides frui*	HepG2 cells	65, 130, 260 μmol/L	Regulation of Nrf2/AMPK/mTOR signaling pathways	Shen et al. (2020)
		Male wild-type mice	50, 75, 100 mg/kg		

### Phenols and Flavonoids

Phenolic compounds are composed of the aromatic rings with one or more hydroxyl groups. They play an important role on oxidative stress in the human by maintaining the balance between oxidants and antioxidants, which are divided into phenolic acids, flavonoids, coumarins, and tannins (Van Hung, 2016). A large number of phenolic compounds in CM have obvious antioxidant capacity, which can reduce the oxidative damage of the liver, such as Lithospermic acid, Chlorogenic acid, Curcumin, Polydatin, and Salvianolic acid C (Chan and Ho, 2015; Koneru et al., 2017; Shi et al., 2016; Wu, C.-T. et al., 2019; Zhong et al., 2016). Yang et al. further found that Chlorogenic acid could reduce the expression of α-SMA, collagen I in the liver tissue and serum TGF-β1 by increasing the mRNA and protein expression of Smad7 and MMP-9, thus alleviating liver fibrosis (Wu, C. et al., 2019). The studies have shown that Curcumin and Polydatin can inhibit lipid accumulation by regulating endoplasmic reticulum stress and the Keap1/Nrf2 pathway (Lee, H.-Y. et al., 2017; Zhao, X.-J. et al., 2018). In addition, Yan et al. demonstrated that Chlorogenic acid could improve liver injury and insulin resistance by inactivating the JNK pathway and inhibiting the autophagy in MAFLD rats (Yan, Hua et al., 2018).

Flavonoids, a part of phenolic compounds, also have significant hepatoprotective effects. For example, Isorhamnetin suppresses the TGF-β/Smad pathway and reduces oxidative stress to alleviate hepatic fibrosis (Yang, J.H. et al., 2016), and Wogonin reduces hepatic fibrosis by regulating the activation and apoptosis of HSCs (Du et al., 2019). Quercetin can effectively alleviate MAFLD, which depends on its regulation of intestinal microbiota imbalance and related gut-liver axis activation (Porras et al., 2017). Hesperidin and Oxylin A have significant anti-hepatoma activity (Mo'men et al., 2019; Wei et al., 2017). In addition, Licochalcone A can increase the expression of antioxidant enzymes by reducing the apoptosis, mitochondrial dysfunction, and reactive oxygen production stimulated by tert butyl peroxide and Acetaminophen, thus protecting APAP-induced hepatotoxicity, which is largely dependent on the antioxidant Nrf2 pathway (Lv et al., 2018). What’s more, rutin has a good protective effect on various acute liver injury induced by carbon tetrachloride, lipopolysaccharide, and mercury chloride (Caglayan et al., 2019; Elsawy et al., 2019; Rakshit et al., 2021).

Bacalin, a kind of flavonoid extracted from *Scutellaria baicalensis*, has significant biological activity, which is widely used in the treatment of liver diseases. The study has shown that bacalin suppresses the production of IL-1β, IL-6, and TNF-α, as well as regulates the TLR4 expression and inhibits the NF-κB activation, protecting the inflammation of chicken’s liver induced by LPS through the negative regulation of inflammatory medium (Cheng et al., 2017). Another study showed that the inhibition of the proliferation, apoptosis, invasion, migration, and activation of HSCs induced by platelet derived growth factor-BB through mir-3595/acsl4 axis is one of the mechanisms of bacalin in anti-hepatic fibrosis (Wu, X. et al., 2018).

**FIGURE 6:**
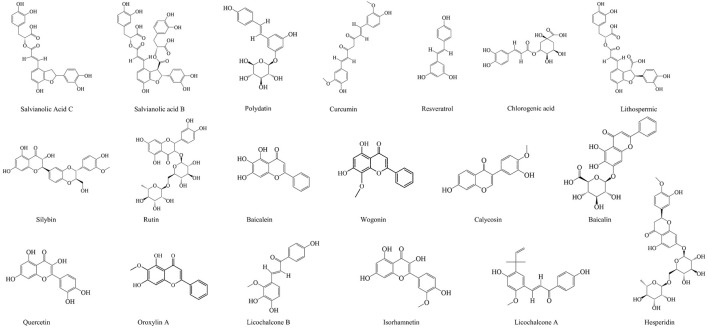
The chemical structures of phenols and flavonoids showing anti-hepatopathy activity.

The specific information of the phenols and flavonoids is shown in Table 3, and the chemical structures of the phenols and flavonoids are shown in Figure 6.

**TABLE 3:** Summary of phenols and flavonoids with significant anti-liver disease activity.

Compounds	Source	The species investigated	Dose	Mechanisms	References
Phenols					
Resveratrol	*Polygonum cuspidatum*	Male C57BL/6J mice	60 mg/kg	Through improving insulin sensitivity and glucose levels	Hajighasem et al. (2018); Zhao et al. (2019a)
		HepG2 cells	20, 50, 100 μM		
		Male Wistar rats	25 mg/kg		
Salvianolic acid B	*Salvia miltiorrhiza*	Male Kunming mice	15, 30 mg/kg	Inhibition of MAPK-mediated P-Smad2/3L signaling	Wu et al. (2019b)
		HSC-T6 cells	25, 50, 100 μM		
		LX-2 cells	25, 50, 100 μM		
Salvianolic Acid C	*Salvia miltiorrhiza*	Male ICR mice	5, 10, 20 mg/kg	By attenuating inflammation, oxidative stress, and apoptosis through inhibition of the Keap1/Nrf2/HO-1 signaling	Wu, et al. (2019c)
Polydatin	*Polygonum cuspidatum*	Male Sprague-Dawley rats	7.5, 15, 30 mg/kg	Through increasing miR-200a to regulate Keap1/Nrf2 pathway, and restoring the antioxidant balance as well as the MMP/TIMP balance	Koneru et al. (2017); Zhao, et al. (2018a)
		BRL-3A cells	10, 20, 40 μM		
		HepG2 cells	10, 20, 40 μM		
		Male C57BL/6 mice	50, 100 mg/kg		
Curcumin	*Curcumin longa*	Pregnant NMRI mice	10 mg/kg	By suppression of oxidative stress-related inflammation *via* PI3K/AKT and NF-kB related signaling	Barandeh et al. (2019); Lee et al. (2017b); Zhong et al. (2016)
		Male Sprague-Dawley rats	200 mg/kg		
		Male C57BL/6 mice	20, 40, 80 mg/kg		
		HSCs	0.5, 1, 2 μM		
Chlorogenic acid	*Oriental Wormwood*	Female Sprague-Dawley rats	50 mg/kg	Inhibition of oxidative stress, JNK pathway and miR-21-Regulated TGF-β1/Smad7 signaling pathway	Shi et al. (2016); Yang et al. (2017)
		Male Sprague-Dawley rats	15, 30, 60 mg/kg		
		HSCs	12.5, 25, 50 mg/ml		
		LX2 cells	20, 40, 80 μg/ml		
Lithospermic acid	*Salvia miltiorrhiza*	Huh-7 cells	5, 10, 20, 40 μg/ml	Reduction of free radicals, restoration of liver functions and inhibition of caspase activity associated with apoptosis	Chan and Ho (2015)
		Male BALB/c mice	50, 100 mg/kg		
Flavonoids					
Hesperidin	*Citrus*	Male Wistar rats	200 mg/kg	Inhibition of free radicals, NF-κB activation and PI3K/Akt pathway, and activation of the Akt pathway	Li et al. (2020b); Mo'men et al. (2019); Pérez-Vargas et al. (2014)
		Male C57BL/6J mice	100, 200, 400 mg/kg		
		Hepatocytes	10, 20 ng/ml		
Licochalcone A	*Licorice Glycyrrhiza*	Nrf2^−/−^ C57BL/6 mice	50, 100 mg/kg	Up-regulation of the Nrf2 antioxidant and sirt-1/AMPK pathway	Liou et al. (2019); Lv et al. (2018)
		HepG2 cells	1.5, 3, 3.7, 6, 12 μM		
		Male C57BL/6 mice	5, 10 mg/kg		
Licochalcone B	*Licorice Glycyrrhiza*	HepG2 cells	40, 80, 120 μM	Inhibition of Caspase 8 and Caspase 9 proteins	Wang et al. (2019b)
Wogonin	*Scutellaria radix*	Male C57BL/6 mice	10, 20, 40 mg/kg	Regulation of hepatic stellate cell activation and apoptosis	Du et al. (2019)
		HSC-T6 cells	1.25 μg/ml		
		LX-2 cells	20 μg/ml		
Quercetin	*Radix Bupleuri*	Male C57BL/6J mice	0.05% (wt/wt)	By ameliorating inflammation, oxidative stress, and lipid metabolism, and modulating intestinal microbiota imbalance and related gut-liver axis activation	Li et al. (2018b); Porras et al. (2017); Yang et al. (2019a); Zhu et al. (2018a)
		Male BALB/c mice	50 mg/kg		
		Raw 264.7 cells	50 μM		
		Male db/db mice	100 mg/kg		
		Male Sprague-Dawley rats	100 mg/kg		
		HepG2 cells	100 μM		
Baicalin	*Scutellariae radix*	Male C57BL/6 mice	15, 30, 60 mg/kg	By regulating the ERK signaling pathway, TLR4-Mediated NF-κB pathway and miR-3595/ACSL4 axis	Cheng et al. (2017); Liao et al. (2017); Wu et al. (2018a)
		HSC-T6 cells	50, 100, 150 μM		
		Young chicken	50, 100, 200 mg/kg		
Baicalein	*Scutellariae radix*	BEL-7402 cells	5, 10 μg/ml	By activating apoptosis and ameliorating P-glycoprotein activity	Li. et al. (2018a)
		BEL-7402/5-FU cells	5, 10 μg/ml		
Rutin	*Forsythia suspensa*	Male db/db mice	60, 120 mg/kg	By interfering with oxidative stress, inflammation and apoptosis, and facilitating signal transduction and activated state of insulin IRS-2/PI3K/Akt/GSK-3β signal pathway	D'Atanasio et al. (2018); Elsawy et al. (2019); Liang et al. (2018); Liu et al. (2017)
		HepG2 cells	8, 16, 32, 64 μg/ml		
		Male albino rats	70 mg/kg		
		Male Sprague Dawley rats	50, 100 mg/kg		
		Male C57BL/6 mice	200 mg/kg		
Calycosin	*Radix astragali*	Male C57BL/6 mice	12.5, 25, 50 mg/kg	By activating farnesoid X receptor	Duan et al. (2017)
Silybin	*Silybum marianum*	Male C57BL/6 mice	105 mg/kg	By reducing oxidative damage to mitochondria, proteins, lipids, and involvement with the NF-κB pathway	Goh et al. (2020); Ou et al. (2018)
		LO2 cells	25, 50 μM		
Isorhamnetin	*/*	Male C57BL/6J mice	50 mg/kg	By inhibiting *de novo* lipogenic pathway, by inhibiting TGF-β/Smad signaling and relieving oxidative stress, inhibiting Extracellular Matrix Formation *via* the TGF-β1/Smad3 and TGF-β1/p38 MAPK Pathways (*via* inhibition of TGF-β1-mediated Smad3 and p38 MAPK signaling pathways.)	Ganbold et al. (2019); Liu et al. (2019a); Yang et al. (2016b)
		LX-2 cells	25, 50, 100 μM		
		HepG2 cells	25, 50, 100 μM		
		Male ICR mice	10, 30 mg/kg		
Oroxylin A	*Scutellaria baicalensis*	Male ICR mice	30 mg/kg	Inhibition of hypoxia inducible factor 1alpha, and activation PKM1/HNF4 alpha	Jin et al. (2018); Wei et al. (2017)
		LO2 cells	10, 20, 40 μM		
		HepG2 cells	6, 8, 10 μM		
		SMMC-7721 cells	15, 20, 25 μM		
		C57BL/6J mice	75 mg/kg		

### Terpenoids

Terpenoids (isoprenoids) are the most abundant chemical compounds in plants (Tholl, 2015), which has a wide range of biological activities, such as anti-inflammation (Kim, T. et al., 2020), anti-depressant (Agatonovic-Kustrin et al., 2020), anti-cancer (Ateba et al., 2018), and so on. Many studies have shown that terpenoids are also widely used in the treatment of liver diseases. Leucodin is a sesquiterpene lactone isolated from *Artemisia capillaris*, which can inhibit the inflammatory response of macrophages, and P2x7R-NLRP3-mediated lipid accumulation in hepatocytes (Shang et al., 2018). Saikosaponin-d is an active component isolated from *Radix Bupleuri*, which can inhibit the COX2 expression through the p-STAT3/C/EBPβ signaling pathway in HCC (Ren et al., 2019). Oleanolic acid (OA) is a kind of triterpenoid widely existing in fruits, vegetables, and herbs. It is liver-specific and can selectively inhibit adipogenesis (Lin, Y.-N. et al., 2018). In addition, OA can regulate antioxidant status, and induce mitochondria-mediated apoptosis and regulate inflammation, which effectively inhibits 7,12-Dimethylbenz[a]anthracene-induced liver cancer (Hosny et al., 2021).

*Rhizoma Alismatis* is a kind of common CM, which is often used in clinic for adverse urination, edema, diarrhea, and so on. Modern studies have shown that many compounds extracted from *Rhizoma Alismatis* have hepatoprotective effects. For example, Alisol A 24-acetate, a natural triterpene extracted from *Rhizoma Alismatis*, can improve NASH by inhibiting oxidative stress, and stimulating autophagy through the AMPK/mTOR signaling pathway (Wu, C. et al., 2018). Meng et al. found that Alisol A 23-acetate could also improve NASH in the mice, which was achieved by the activation of X-like receptor (Meng et al., 2017). Futhermore, Meng et al. found that Alisol A 23-acetate activated FXR to induced the phosphorylation of STAT3 and the expression of its target genes, Bcl-xl and SOCS3. And it reduced the expression of the liver uptake transporter NTCP, and bile acid synthases CYP7A1 and Cyp8b1, as well as increased the expression of the outflow transporters BSEP and MRP2, reducing the hepatic bile acid deposition, which achieved the protective effect on CCl_4_-induced hepatotoxicity in the mice (Meng et al., 2015).

**FIGURE 7:**
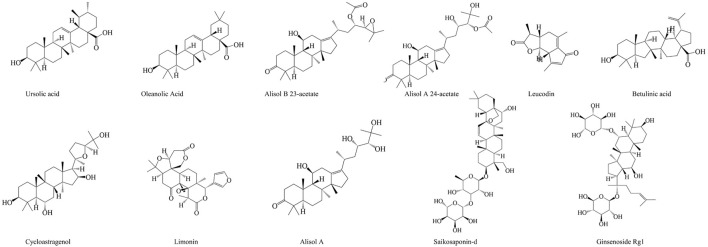
The chemical structures of terpenoids showing anti-hepatopathy activity.

The specific information of the terpenoids in the treatment of liver diseases is shown in Table 4, and the chemical structures of the terpenoids with therapeutic effects on liver diseases are shown in Figure 7.

**TABLE 4:** Summary of terpenoids with significant anti-liver disease activity.

Compounds	Source	The species investigated	Dose	Mechanisms	References
Betulinic acid	*Betula pubescens*	Male C57BL/6J mice	15, 30, 60, 150 mg/kg	Through the YY1/FAS, MAPK/ERK and PI3K/AKT/mTOR signaling pathway	Liu et al. (2019b); Liu et al. (2019c); Mu et al. (2020)
		SMMC-7721 cells	2.5, 5, 10, 20, 40 μM		
		HepG2 cells	2.5, 5, 10, 20, 40 μM		
Saikosaponin-d	*Radix Bupleuri*	SMMC-7721 cells	2.5, 5, 10 μg/L	Through SENP5- Dependent Inhibition of Gli1 SUMOylation Under Hypoxia, and p-STAT3/C/EBPβ signaling	Ren et al. (2019); Zhang et al. (2019)
		HepG2 cells	2.5, 5, 10 μg/L		
Cycloastragenol	*Astragali Radix*	HepG2 cells	12, 25, 50 μM	By activating farnesoid X receptor signaling	Gu et al. (2017)
		Female C57BL/6 mice	100 mg/100 g diet		
Limonin	Citrus fruit and plants	Male Wistar rats	100 mg/kg	By activating Nrf2 antioxidative pathway and inhibiting NF-κB inflammatory response and TLR-signaling pathway	Mahmoud et al. (2014); Yang et al. (2020b)
		L-02 cells	10, 25, 50 μM		
		Male C57BL/6 mice	40, 80 mg/kg		
Oleanolic acid	*Forsythia suspensa*	Male Swiss albino mice	75 mg/kg	Through induction of mitochondrial-mediated apoptosis and autophagy, and inhibition of Liver X Receptor Alpha and Pregnane X Receptor	Hosny et al. (2021)
		EAC cells	9.32 μM		
		HepG2 cells	10, 20, 32.58, 27.56 μM		
		SMMC-7721 cells	10, 30, 60 μmol/L		
Ginsenoside Rg1	*Panax ginseng*	Male C57BL/6 mice	15, 30, 60 mg/kg	By activating Nrf2 signaling pathway	Ning et al. (2018)
Ursolic acid	*Forsythia suspensa*	C57BL/6 mice	40 mg/kg	Through RhoA-related signaling pathways, and inhibition of interactive NOX4/ROS, RhoA/R and CASP3	Gan et al. (2018a); Ma et al. (2021); Wan et al. (2019); Wan et al. (2020)
		HepG2 cells	10 μM		
		Male Kunming mice	20, 40, 80 mg/kg		
		Sprague–Dawley rats	40 mg/kg		
Alisol A	*Rhizoma Alismatis*	C57BL/6 mice	100 mg/kg	Through the AMPK/ACC/SREBP-1c pathway	Ho et al. (2019)
Alisol B 23-acetate	*Rhizoma Alismatis*	Male C57BL/6 mice	10, 15, 20, 30, 40, 60 mg/kg	Regulation of the FXR and STAT3 signaling pathway	Meng et al. (2015); Meng et al. (2017)
Leucodin	*Artemisia capillaris*	HepG2 cells	1, 5 μM	Through the P2x7 receptor pathway	Shang et al. (2018)

### Alkaloids

Alkaloids are an important class of natural products, which have a wide range of biological activities, and have been used in folk medicine for many years (Stöckigt et al., 2011). We are surprised to find that alkaloids play an important role in the treatment of liver diseases. Matrine and Oxymatrine are the main active substances extracted from the roots of *Sophora flavescens*, and are widely used (Yuan et al., 2010). They have significant biological activities against MAFLD, liver injury, and liver cancer (Gao et al., 2018; Shi et al., 2020; Wei et al., 2018; Xu et al., 2018; Zhang, H. et al., 2020). Ligustrazine is an alkaloid extracted from *Ligusticum chuanxiong*. It not only activates Nrf2 to inhibit hepatic steatosis, but also induces the apoptosis and autophagy of hepatoma cells to exert an anti-hepatoma effect (Cao et al., 2015; Lu et al., 2017). And coptisine exerts an anti-hepatoma effect by activating the 67 kDa laminin receptor/cGMP signal to induce the apoptosis of human hepatoma cells, and the proliferation and migration of HCC cells (Chai et al., 2018a; Zhou et al., 2018).

**FIGURE 8:**
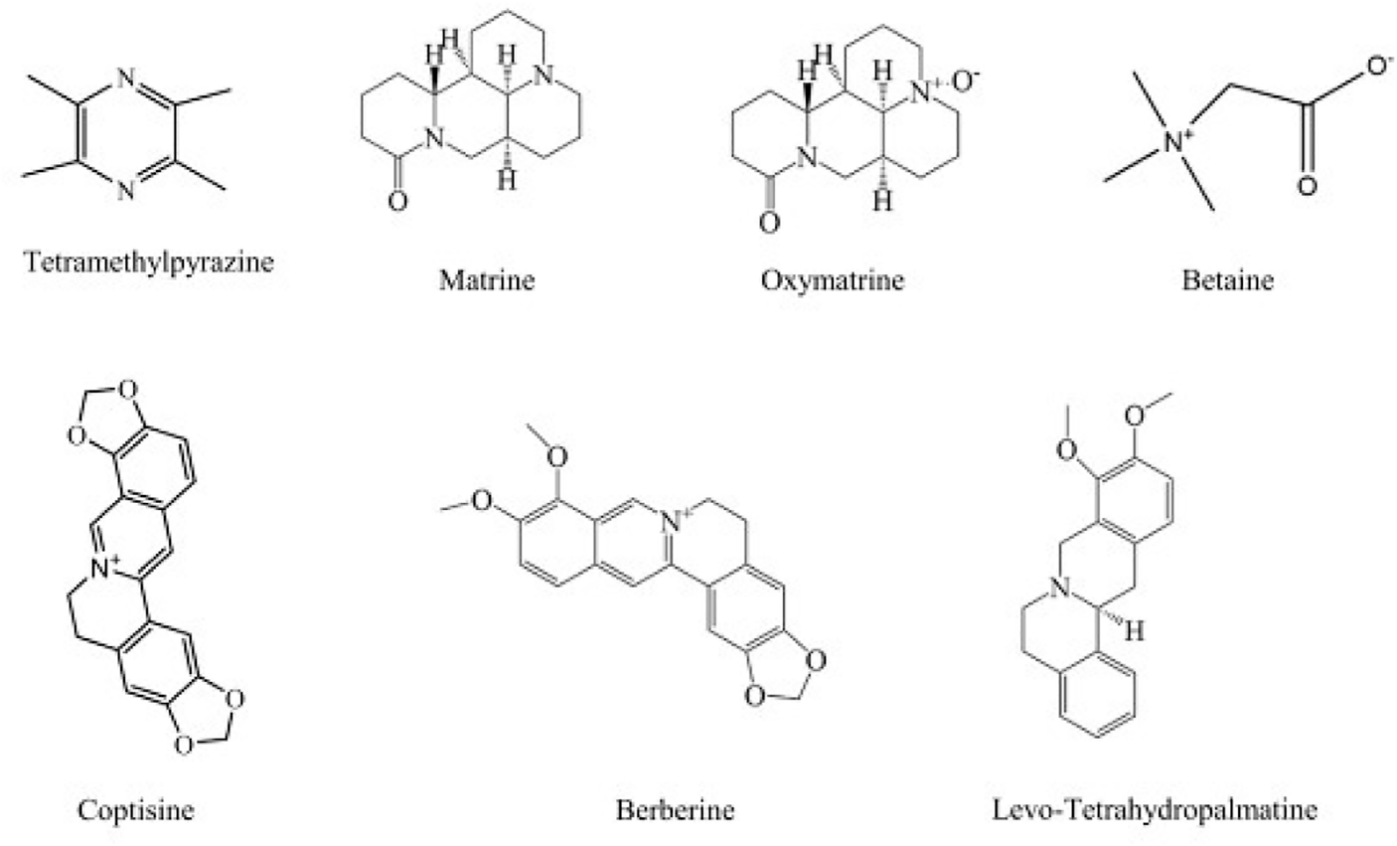
The chemical structures of alkaloids showing anti-hepatopathy activity.

The specific information of various alkaloids in the treatment of liver diseases is shown in Table 5. In addition, the chemical structure formulas are shown in Figure 8.

**TABLE 5:** Summary of alkaloids with significant anti-liver disease activity.

Compounds	Source	The species investigated	Dose	Mechanisms	References
Tetramethylpyrazine	*Ligusticum chuanxiong Hort*	Male Sprague-Dawley rats	50, 100, 200 mg/kg	Through PDGF-bR/NLRP3/caspase1 pathway to reduce liver inflammation, and exerts antitumor effects by inducing apoptosis and autophagy in hepatocellular carcinoma, and inhibition of hepatic steatosis by activating the Nrf2 signaling pathway	Cao et al. (2015); Lu et al. (2017); Wu et al. (2015)
		Human HCC HepG2 cells	50, 100, 200 μM		
		Male BALB/c nude mice	50, 100, 150 mg/kg		
		Male ICR mice	100 mg/kg		
		Human LO2 hepatocytes	20 μM		
Coptisine	*Rhizoma Coptidis*	Kunming mice	37.5, 150 mg/kg	Through up-regulating expression of miR-122, and activating 67-kDa laminin receptor/cGMP signaling	Chai et al. (2018a); Chai et al. (2018b); Zhou et al. (2018)
		HepG2 cells	12.5, 25, 50, 100 μg/ml		
		L02 cells	25 μg/ml		
		SMMC7721 cells	12.5, 25, 50, 100 μM		
		Male BALB/c nude mice	150 mg/kg		
		HepG2 cells	25 μg/ml		
		Huh7 cells	25 μg/ml		
Matrine	*Sophora flavescens*, *Sophora subprostrata*	Male C57BL/6J mice	0.5, 2.5, 10 mg/kg	Regulation of SERCA pathway, and inhibition of mitophagy, PINK1/Parkin pathways and Notch signaling pathway	Gao et al. (2018); Wei et al. (2018)
		L02 cells	200, 400, 800 μM		
		HepG2 cells	1, 5 nM		
		Huh7 cells	1, 5 nM		
Betaine	*Lycium chinensis*	Male Sprague-Dawley rats	20 g/kg	Regulation of oxidative stress, inflammation, apoptosis, autophagy and Akt/mTOR signaling	Abu Ahmad et al. (2019); Veskovic et al. (2019)
		Male C57BL/6 mice	1.5% (w/v)		
Berberine	*Rhizoma Coptidis*	Male C57BL/6 mice	2, 5 mg/kg	Inhibition of oxidative stress, hepatocyte necrosis, inflammatory response, and AKT-mTOR-S6K signaling pathway	Li. et al. (2018a); Zhao et al. (2018b)
		MIHA cells	10, 20, 100 μM		
		HepG2 cells	10, 20 μM		
Oxymatrine	*Sophora alopecuroides*	Male Sprague-Dawley rats	30, 60, 120 mg/kg	Activation of Nrf2/HO-1, regulation of miR-182, and modulation of TLR4-dependent inflammatory and TGF-β1 signaling pathways	Xu et al. (2018); Zhang et al. (2020b); Zhao et al. (2016)
		BMDMs	1.0 mg/ml		
		HSC-T6 cells	250, 500, 1000 μg/ml		
		Male C57BL/6 mice	120 mg/kg		
		Wistar male rats	80 mg/kg		
Levo-tetrahydropalmatine	*Corydalis yanhusuo*	Male C57 mice	20, 40 mg/kg	Modulation of PPARγ/NF-κB, TGF-β1/Smad and TRAF6/JNK signaling pathway	Yu et al. (2021); Yu et al. (2018)
		LX-2 cells	34.01 μmol/L		
		Male Balb/c mice	20, 40 mg/kg		

### Other Bioactive Ingredients

**FIGURE 9:**
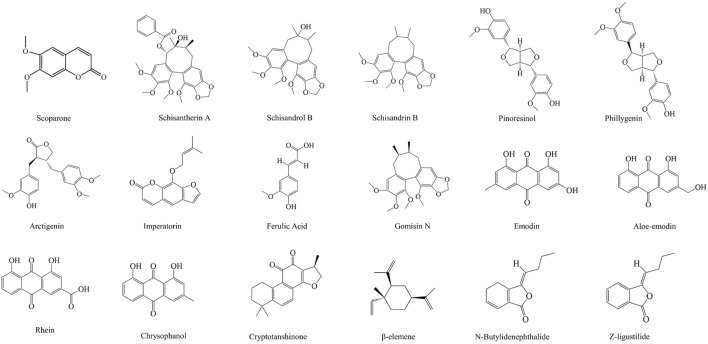
The related chemical structures of other anti-hepatopathy bioactive components.

In addition to the above compounds, many compounds have the activities of anti-liver diseases, including phenylpropanoids (such as simple phenylpropanoids, coumarins, and lignans), anthraquinones, and volatile oils. Some lignans extracted from CM have been proved to have the effects on improving liver diseases. For example, Gomisin N extracted from *Schisandra chinensis* not only has protective effects on endoplasmic reticulum stress-induced hepatic steatosis, but also alleviates the liver injury caused by ethanol by improving lipid metabolism and oxidative stress (Jang et al., 2016; Nagappan et al., 2018). Futhermore, Arctigenin can inhibit the proliferation of HepG2 cells and block the autophagy cells that lead to the accumulation of sequestosome 1/p62, so as to achieve the therapeutic effects on liver cancer. It will become a new drug for the autophagy research and cancer chemoprevention. It is worth noting that many anthraquinones in *Rhubarb* have good activities of anti-liver diseases, including chrysophanol, emodin, rhein, and aloe emodin (Bai et al., 2020; Dong et al., 2017; Kuo et al., 2020; Li, Y. et al., 2019). Cryptotanshinone, the main anthraquinone extracted from *Salvia miltiorrhiza* Bunge, can protect liver by activating the AMPK/SIRT1 and Nrf2, and inhibiting CYP2E1 to inhibit adipogenesis, oxidative stress, and inflammation (Nagappan et al., 2019). Other bioactive components against liver diseases are shown in Table 6. In addition, the related chemical structures are also shown in Figure 9.

**TABLE 6:** Summary of other bioactive ingredients with significant anti-liver disease activity.

Compounds	Source	The species investigated	Dose	Mechanisms	References
Phenylpropanoids					
Ferulic Acid	*Angelica sinensis*	Male Swiss albino mice	50, 100 mg/kg	Upregulation of Nrf2/HO-1 signaling, and inhibition of TGF-β/smad signaling pathway, and modulation of the gut microbiota composition	Ma et al. (2019); Mahmoud et al. (2020); Mu et al. (2018); Roghani et al. (2020)
		Male ApoE^−/−^ mice	30 mg/kg		
		Male Wistar rats	10, 25, 50 mg/kg		
		LX-2 cells	5, 15, 30 μM		
Phillygenin	*Forsythia suspensa*	LX2 cells	12.5, 25, 50 μM	Through TLR4/MyD88/NF-κB signaling pathway	Hu et al. (2020b)
Arctigenin	*Arctium lappa*	HepG2 cells	10 μM	Through autophagy inhibition in hepatocellular carcinoma cells	Okubo et al. (2020)
		MCF-7 cells	10 μM		
Imperatorin	*Angelica dahurica*	Male C57BL/6 mice	50, 100 mg/kg	By stimulating the SIRT1-FXR pathway	Gao et al. (2020)
		Hepatocytes	5, 10 μM		
Pinoresinol	*Forsythiae Fructus*	Male ICR mice	25, 50, 100, 200 mg/kg	Through inhibition of NF-κB and AP-1	Kim et al. (2010)
Schisandrol B	*Schisandra sphenanthera*	Male C57BL/6 mice	12.5, 50, 200 mg/kg	Inhibition of CYP-mediated bioactivation and regulation of liver regeneration	Jiang et al. (2015)
Schisantherin A	*Schisandra sphenanthera*	Male C57BL/6 mice	25, 50, 100, 200, 400, 800 mg/kg	Inhibition of mitogen-activated protein kinase pathway	Zheng et al. (2017)
Schisandrin B	*Schisandra sphenanthera*	Male Wistar rats	25, 50 mg/kg	Regulation of Nrf2-ARE and TGF-β/smad signaling pathways	Chen et al. (2017)
		HSC-T6 cells	5, 10, 30 μM		
Gomisin N	*Schisandra sphenanthera*	Male C57BL/6N mice	5, 20 mg/kg	Through ameliorating lipid metabolism, oxidative Stress and ER stress	Nagappan et al. (2018); Yun et al. (2017)
		HepG2 cells	10, 50, 100 μM		
		C57BL/6 mice	1, 30 mg/kg		
Scoparone	*Artemisia capillaris*	AML12 cells	200 mM	By regulating the ROS/P38/Nrf2 axis, PI3K/AKT/mTOR pathway, and TLR4/NF-κB signaling pathway	Liu et al. (2020b)
		RAW264.7 cells	25, 50, 100, 200 mM		
		Male C57BL/6 J mice	20, 40, 80 mg/kg		
Anthraquinones					
Chrysophanol	*Rheum palmatum*	HSC-T6 cells	30 mM	By regulating endoplasmic reticulum stress and ferroptosis	Kuo et al. (2020)
Emodin	*Rheum palmatum*	Male BALB/c nude mice	15, 25, 30, 50, 60 mg/kg	By regulating VEGFR2, miR-34a, AMPK with Hippo/Yap signalling pathway, MAPK, PI3K/AKT signaling pathways, and inhibiting the TLR4 signaling pathway and epithelial-mesenchymal transition and transforming growth factor-β1	Bai et al. (2020); Ding et al. (2018a); Lee et al. (2020); Lin et al. (2016); Liu et al. (2018)
		HepG2 cells	3, 10, 30, 100 μM		
		SK-Hep-1 cells	30 μM		
		Male C57B/6 mice	10, 30 mg/kg		
		Male Sprague-Dawley rats	10, 20, 40 mg/kg		
		RAW264.7 cells	15, 30, 60 μg/ml		
		Male Balb/c mice	20, 40, 80 mg/kg		
		SMMC-7721 cells	25, 50, 100 μM		
Rhein	*Polygonum multiflorum*	Male Sprague-Dawley rats	10, 30, 1000 mg/kg	Through regulating the Fas death pathway and the mitochondrial pathway, and promoting bile acid transport and reduce bile acid accumulation	Li et al. (2019c); Xian et al. (2020)
		L02 cells	25, 50, 100 μM		
Aloe-emodin	*Rheum palmatum*	HepG2 cells	1, 15, 30 μM	Regulation of the Fas death pathway and the mitochondrial pathway, and inhibition of multidrug resistance protein 2	Dong et al. (2017); Liu et al. (2020b)
		Male and female Kunming mouse	0.8, 1.6 g/kg		
		HL-7702 cells	5, 10, 20, 40 μM		
Cryptotanshinone	*Salvia miltiorrhiza*	Male C57BL/6 mice	20, 40 mg/kg	Inhibition of MAPKs phosphorylation regulated by TAK1, and activation of AMPK/SIRT1 and Nrf2 signaling pathways	Jin et al. (2014); Nagappan et al. (2019)
		HepG2 cells	2.5, 5 μM		
		AML-12 cells	2.5, 5 μM		
Volatile oil					
Z-ligustilide and n-Butylidenephthalide	*Angelica tenuissima*	MaleC57BL/6mice	10, 50 mg/kg	Inhibition of fatty acid uptake and esterification	Lee et al. (2019)
		HepG2 cells	10, 50, 100 μg/ml		
Butylidenephthalide	*Angelica sinensis*	HSC-T6 cells	15, 25, 35 μg/ml	Reduction of EMT, decreasing inflammatory reaction, and liver cell proliferation	Chuang et al. (2016)
		Male Wistar rats	15, 80 mg/kg		
Ligustilide	*Angelica sinensis*	Male Sprague-Dawley	10, 20, 40 mg/kg	Promotion of phosphorylation of Nrf2 and AMPKa1	Guo et al. (2021)

### Toxicity

After the above discussion, it is not difficult to find the important position of CM in the treatment of liver diseases. As we all know, CM is a relatively safe class of drugs, but we can’t ignore its toxic and side effects on the liver when we use CM to treat liver diseases. The studies have found that some CM show certain hepatotoxicity. For example, *Rhubarb* extract had a certain protective effect on the rats with chronic renal failure, but the incidence of mild hepatotoxicity was also observed in normal rats (Wang et al., 2009). *Ginkgo biloba* extract induced DNA damage by inhibiting the topoisomerase II activity in human hepatocytes (Zhang et al., 2015). Interestingly, the hepatotoxicity of some CM comes from their own hydrolysates. For example, after the intragastric administration of *Sophora flavescens* extract to the rats, kurarinone glucosides was hydrolyzed into Kurarinone in liver cells, which eventually led to the lipid accumulation and liver injury through a series of actions (Jiang, P. et al., 2017). In addition, the use of herbal products is also a crucial cause of acute liver injury. It has been reported that a 68-year-old woman suffered from acute liver injury caused by aloe, after stopping taking aloe, her liver functions returned to normal levels (Parlati et al., 2017). It is worth noting that the first case of autoimmune hepatitis caused by turmeric supplements has been reported (Lukefahr et al., 2018).

The dosage of CM is often closely related to hepatotoxicity. In order to study the hepatotoxicity of *Cortex Dictamni*, fan et al. used its water extract and alcohol extract to carry out the toxicity experiments *in vivo* and *in vitro*. The results showed that high dose of water extract and alcohol extract significantly increased the levels of ALT and AST, absolute and relative liver weight, and the liver-to-brain ratio, and the histological examination of the liver showed the cell enlargement and nuclear contraction. *In vitro* cell experiment also showed that water extract and alcohol extract reduced the cell viability in a dose-dependent manner (Fan et al., 2018). A single oral dose of 60 g/kg *Cortex Dictamni* ethanol extract for 24 h resulted in severe hepatocyte necrosis in mice, and the induced liver injury showed a dose and time-dependent manner (Huang et al., 2020). Saikosaponins, a major bioactive component extracted from *Radix Bupleuri*, enhances the CYP2E1 expression in a dose and time-dependent manner, and induces oxidative stress *in vivo* and *in vitro*, leading to liver injury in mice (Li et al., 2017). In another study, the rats were fed with 300, 1250 and 2500 mg kg^−1^·D^−1^ *Radix Scutellariae Baicalensis* ethanol extract for 26 weeks. It was found that the liver tissues of the rats in the high-dose group showed some inflammatory changes mainly characterized by leukocyte infiltration. In addition, there were also some changes in the levels of glucose, electrolyte, and lipid (Yi et al., 2018). It can be seen that the hepatotoxicity of many CM are closely related to the dosage.

In addition, the abuse of CM without the guidance of doctors is also the source of toxic reactions. Because traditional Chinese medical science thinks that “toxicity” refers to the biases of drugs, the toxic components of CM are often the effective components for treating diseases. The key to judging whether CM is toxic or non-toxic is to see whether it is used according to the syndrome. As long as the treatment is besed on the syndrome, toxic drugs are also safe. If the treatment is not for the syndrome, non-toxic drugs may be harmful. It is worth noting that there are also some CM products considered non-toxic or low toxic, which have obvious toxicological effects on different organs in animal and human models (Liu, R. et al., 2020). So it is a great problem to control the toxic and non-toxic boundaries reasonably, and every traditional medical scholar should make efforts to do so.

### Clinical Trials

Most drugs for anti-liver diseases used in clinic are CM compounds, and less clinical research and application involve only one CM or one compound. Table 7 shows some CM (excluding CM compounds) used in the clinical treatment of liver diseases. The purpose is to improve the richness of clinical medication, so that more CM with potential and significant therapeutic effects can be noticed.

**TABLE 7:** Some Chinese medicine are used to treat liver diseases in clinic.

CM or its compounds	disease	Subject	Study design	Treatment groups	Length	Clinical outcome	References
Turmeric supplementation (Combined use of chicory seeds)	MAFLD	92 patients (aged 20–60 years)	Double-blind, randomized, controlled clinical trial	Control group: placebo	12 weeks	Significantly decreased serum alkaline phosphatase and increased serum HDL-C	Ghaffari et al. (2019)
				Experimental group: turmeric supplementation (3 g/d, TUR); Chicory seed supplementation (9 g/d, CHI); Turmeric and chicory seed supplementation (3 g/d, TUR + CHI)			
Curcuminoids supplementation	MAFLD	55 patients	Double-blind, randomized, placebo-controlled trial	Control group: placebo capsules	8 weeks	Improved the severity of MAFLD; serum concentrations of TNF-α, MCP-1 and EGF were improved	Saberi-Karimian et al. (2020)
				Experimental group: 500 mg curcuminoids (plus 5 mg piperine to increase intestinal absorption)			
Curcumin (amorphous dispersion formulation)	MAFLD	80 cases	Randomized double-blind placebo-controlled trial	Control group: placebo	8 weeks	The liver fat content, biochemical parameters and anthropometry were significantly improved in patients with MAFLD	Rahmani et al. (2016)
				Experimental group: 500 mg/day equivalent to 70 mg curcumin			
Curcumin supplementation	LC	70 patients (aged 20–70 years)	Randomized, double-blind, placebo-controlled trial	Control group: placebo	3 months	MELD(i), MELD, MELD-Na and Child-Pugh scores decreased significantly	Nouri-Vaskeh et al. (2020b)
				Experimental group: 1000 mg/day curcumin			
Curcumin	LC	70 cases (aged 20–70 years)	Randomized double-masked placebo-controlled trial	Control group: placebo	12 weeks	The total score and most of the CLDQ, physical and mental health scores and most of the SF-36 areas were significantly improved, and the LDSI2.0 domain was significantly decreased	Nouri-Vaskeh et al. (2020a)
				Experimental group: 1000 mg/day curcumin			

Resveratrol	MAFLD	60 subjects	Double-blind, randomized, placebo-controlled trial	Control group: placebo	3 months	Significantly reduced aspartate aminotransferase (AST), glucose, LDL-C, total cholesterol; reduced the levels of tumour necrosis factor-alpha, cytokeratin 18 and fifibroblast growth factor 21	Chen et al. (2015)
				Experimental group: 150 mg resveratrol twice daily			
*Portulaca oleracea* L. hydroalcohoic extract	MAFLD	74 patients	Randomized, double-blind clinical trial	Control group: placebo capsules	12 weeks	The levels of alanine aminotransferase (ALT), aspartate transaminase, γ-glutamyltransferase, fasting blood glucose, insulin resistance, triglyceride and LDL-C were significantly reduced	Darvish Damavandi et al. (2021)
				Experimental group: 300 mg purslane extract			
*Portulaca oleracea* L. seeds	MAFLD	Sixty eligible individuals (12 men and 48 women)	Randomized controlled clinical trial	Control group: low-calorie diet	8 weeks	Reduced fasting blood glucose, total cholesterol, and LDL-C	Gheflati et al. (2019)
				Experimental group: 10 g/day of purslane seeds and low-calorie diet			
Hesperidin (Combined use of flaxseed)	MAFLD	One hundred eligible patients	Randomized, controlled, clinical trial	Control group: lifestyle modification program	12 weeks	The levels of ALT, insulin resistance, insulin sensitivity index, fasting blood glucose and fatty liver index decreased significantly	Yari et al. (2021)
				Experimental group: lifestyle modification program with 30 g whole flaxseed powder; lifestyle modification program with 1 g hesperidin supplementation; lifestyle modification program with combination of 30 g flaxseed and 1 g hesperidin			
*Artemisia annua* L. Extract	Nonalcoholic liver dysfunction	79 subjects	Randomized, double-Blind, placebo-controlled	Control group: placebo	4 weeks	Levels of AST and ALT were significantly reduced, and scores on the multidimensional fatigue scale were reduced, significantly enhancing liver function and health	Han et al. (2020)
				Experimental group: powdered-water extract of *Artemisia annua*			
Silymarin	NASH	78 patients	Randomized, double-blind, placebo controlled trial	Control group: placebo	12 months	After 48 weeks of treatment, the MAFLD activity score (NAS) decreased by at least two points, fibrosis stage improved, baseline changes, serum ALT and AST decreased	Navarro et al. (2019)
				Experimental group: proprietary standardized silymarin preparation 420 mg or 700 mg			
Silymarin	NASH	99 patients	Randomized, double-blind, placebo-controlled trial	Control group: placebo	48 weeks	The fibrosis was reduced and the ratio of AST to platelet index was also significantly decreased	Wah Kheong et al. (2017)
				Experimental group: Silymarin (three times daily)			

Single extract or chemical component of CM showed good activity of anti-liver diseases in clinical research. *Artemisia annua* L. extract can improve the liver function in the patients with mild to moderate nonalcoholic liver dysfunction, and no obvious adverse reactions were observed in all subjects (Han et al., 2020). Futhermore, *Portulaca oleracea* extract can improve liver enzyme, blood lipid, and blood glucose in the patients with MAFLD (Darvish Damavandi et al., 2021). It is worth noting that *Carcuma longa* has a wide range of clinical applications, with a large number of clinical data, suggesting its position in the clinical treatment of liver diseases. To assess the effect of *Carcuma longa* on MAFLD, 92 MAFLD patients aged 20–60 years were enrolled in a 12-week study. The results showed that *Carcuma longa* supplement was very useful in controlling MAFLD-related risk factors (Darvish Damavandi et al., 2021). Curcumin, the main active component of *Curcuma longa*, can increase the serum inflammatory cytokine levels in the patients with MAFLD, which may be partly dependent on the anti-steatosis effect (Saberi-Karimian et al., 2020). In addition, curcumin can improve the quality of life of the patients with liver cirrhosis (Nouri-Vaskeh et al., 2020a). Although the clinical application of *Curcuma longa* has surpassed other CM against liver diseases, it still fails to solve the problem of its optimal dosage, and the molecular mechanisms on treating liver diseases is unclear. More importantly, in view of the widespread use of *Curcuma longa*, we need larger, more impartial and high-quality controlled randomized trials to conduct a deeper evaluation.

In the future, more clinical experiments should be studied, which makes more CM into clinical application, and even go to the international stage. There are still many deficiencies in the current clinical research. First, the dosage is single and the sample size is small, which is not good for screening the best treatment dose. Secondly, the existing clinical experiments mainly focus on the study of MAFLD, but there are many kinds of liver diseases. In the future, the research can be expanded to make more patients with liver diseases benefit from CM. Finally, the mechanisms of many CM (especially CM compounds) used in the treatment of liver diseases are not clear. We should further explore the mechanism of action of CM, making its fuzzy mechanism clearer and letting more people accept it.

## Conclusion and Perspectives

In conclusion, CM can prevent and treat liver diseases through many ways, including regulating lipid metabolism, anti-liver injury (such as CCl_4_, H_2_O_2_, alcohol, and drug damage), anti-oxidant stress (including reducing ROS, increasing SOD, GSH and CAT content, and regulating Nrf2 and other related pathways), regulating bile acid metabolism (including regulating the excreted and ingested receptors), regulating the immune system, anti-hepatitis virus, and anti-liver cancer. In terms of the current situation, a large number of studies have proved the potential of CM in the treatment of liver diseases. However, the resources of CM are huge, and it is probably known that the effective CM for liver diseases are only one corner of the iceberg. More tasks need the joint efforts of all traditional medicine scholars. In addition, a large part of the current research has not only been focused on the study of efficacy, but also the expression level of genes and proteins. But it is not enough, and more new methods should be explored, such as using multi-group analysis (metabolomics, proteomics), so as to promote the progress of CM in the treatment of liver diseases.

It is worth noting that there is also relevant evidence that the new technology of CM combined with other preparations can greatly enhance the therapeutic effects on liver diseases. For example, due to the characteristics of unstable chemical structure, low bioavailability, easy oxidation, and UV degradation, the toxic effect of curcumin on hepatoma cells is limited. Therefore, Kong et al. used curcumin loaded mesoporous silica nanoparticles, and found that the complex had better antioxidant activity than curcumin alone, as well as significantly enhanced the cytotoxic effect on hepatoma cells (Kong et al., 2019). Another study showed that curcumin liposome had a greater inhibitory effect on the growth and apoptosis of cancer cells (Feng et al., 2017). But these studies are still very few, which should be increased later.

This paper lists and elaborates the active ingredients of some CM against liver diseases, such as polysaccharides, glycosides, phenols, flavonoids, terpenoids, alkaloids, etc. We found the research on the mechanism of action of each ingredient was relatively single, and CM showed the joint action of multi-component and multi-target in the treatment of liver diseases. Therefore, screening more effective components and studying their molecular mechanisms should be greatly strengthened. For example, recent studies have shown that iron is essential for life, but excessive iron may be cytotoxic, which may lead to cell death and some diseases (Bogdan et al., 2016; Nakamura et al., 2019). In addition, in the previous discussion, we also know that the gut microbiota plays an important role in the treatment of liver diseases. Therefore, it is suggested that we can refer to these relevant mechanisms in the future research of CM on treating liver diseases.

CM, including Tibetan medicine, has shown good effects of anti-liver diseases (Li, Qi et al., 2018; Fu et al., 2020), which is indispensable in the treatment of liver diseases. This paper is a comprehensive review of CM and the related compounds, toxicology, and clinical research, which is aimed to provide scientific and effective references for the treatment of liver diseases, and to better use and develop the treasure of CM.

## Abbreviations

ALD, alcoholic liver disease; AMPK, adenine monophosphate activated protein kinase; ARE, antioxidant responsive element; BAs, bile acids; CM, Chinese medicine; CYP, cytochrome P450; FL, fatty liver; FSE, Forsythiae Fructuse water extract; GSH, glutathione; HBV, hepatitis B virus; HCC, hepatocellular carcinoma; HCV, hepatitis C virus; HSCs, hepatic stellate cells; IL-1β, interleukin-1β; IL-6, interleukin-6; KCs, kupffer cells; LC, liver cirrhosis; LSECs, liver sinusoidal endothelial cells; MAFLD, metabolic associated fatty liver disease; NAFL, non-alcoholic fatty liver; NASH, non-alcoholic steatohepatitis; NF-κB, nuclear factor kappa-B; NK, natural killer; Nrf2, nuclear factor-erythroid 2-related factor 2; ROS, reactive oxygen species; RSM, Radix Salvia Miltiorrhiza; SOD, superoxide dismutase; TC, total cholesterol; TG, triglyceride; TGF-β, transforming growth factor-β; TNF-α, tumor necrosis factor-α.
